# Generation of topologically defined linear and cyclic DNA bottle brush polymers *via* a graft-to approach†

**DOI:** 10.1039/d5py00082c

**Published:** 2025-05-01

**Authors:** Nicholas G. Pierini, Wynter A. Paiva, Owen C. Durant, Aubrianna M. Dobbins, Ben B. Wheeler, Matthew E. Currier, James Vesenka, Nathan J. Oldenhuis

**Affiliations:** a Department of Chemistry, College of Engineering and Physical Science, University of New Hampshire 23 Academic Way Parsons Hall Durham NH 03824 USA nathan.oldenhuis@unh.edu; b School of Molecular and Physical Sciences, University of New England 11 Hills Beach Road Biddeford ME 04005 USA

## Abstract

Herein, we report a graft-to approach for synthesizing linear and circular double-stranded DNA (dsDNA) bottlebrush polymers (BBPs). Using a bioreactor, plasmid DNA (pDNA) serves as an inexpensive and abundant source of circular, biodegradable, and unimolecular polymers. pDNA is easily converted to the linear isoform through enzymatic restriction, providing access to polymeric backbones with distinct topological states. DNA is grafted with polyethylene glycol monomethyl ether chloroethylamines (mPEGCEA) to yield DNA BBPs. Importantly this PEGylation occurs rapidly under ambient conditions in aqueous buffer. By varying the molecular weight of mPEGCEA (*M*_w_ = 750, 2000, 5000 Da) and the concentration relative to μmol of nucleotides, different brush arm densities and lengths were achieved with both linear and macrocyclic DNA backbones. Analysis of the DNA BBPs was achieved through agarose gel electrophoresis, which showed graft densities of up to ~68% and ~74% for linear and ring DNA respectively. The grafting process does not alter base pairing or circularity as determined using atomic force microscopy. Shear rheology was used to compare the mechanical response of 1% wt/wt solutions of the ring and linear DNA BBPs to their un-alkylated forms. Linear DNA BBPs exhibited a lower shear modulus *versus* linear DNA, which is expected due to the increased persistence length and decreased ability to interpenetrate associated with the attachment of polymer arms. However, the circular DNA BBPs exhibited a universally higher shear modulus *versus* the un-alkylated sample suggesting an increase in interchain interaction *via* addition of polymer arms. Finally, the increased steric encumbrance of the DNA BBPs slows enzymatic degradation, potentially providing a general method to increase stability of DNA constructs towards nuclease.

## Abstract 2

Plasmid-derived DNA bottlebrush polymers with linear and circular topologies are synthesized *via* aqueous grafting of PEG. These constructs exhibit tunable mechanics, enhanced nuclease resistance, and preserve DNA structure and circularity.

## Graphical abstract

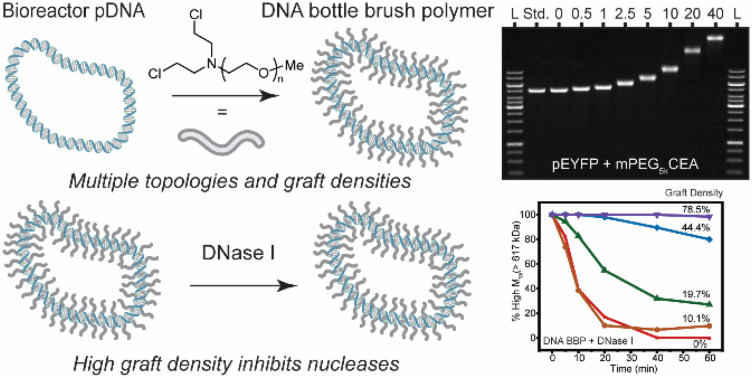

## Introduction

Bottle brush polymers (BBPs) are complex macromolecules featuring grafted side chains attached to a polymeric backbone.^1–3^ By manipulating side chain length, density, and composition, BBPs can achieve remarkable rigidity, low entanglement and viscoelastic moduli, controllable self-assembly, and integrated structural functionality.^1–3^ Their unique architecture has enabled a wide variety of applications, including biomimetic materials, soft-elastomers, and drug-delivery. Recently, BBPs with cyclic backbones have become increasingly popular synthetic targets due to their altered molecular and mechanical properties such as a smaller hydrodynamic radius, lower intrinsic viscosities, lower inter-chain entanglements, increased chemical stability, and higher glass transition temperatures.^4–11^

Typically, the synthesis of the backbone is constrained through the method used to append the sidechains, *e.g.* graft-to, graft-from, or graft-through.^11–13^ While each of the aforementioned methods has been employed to make cyclic BBPs, they commonly suffer from low yields, high dispersity, linear impurities, and poor biodegradability due to the inherent challenges associated with making any cyclic polymers.^12,14–17^ Low dispersity and lack of linear impurities are incredibly important to achieve when investigating cyclic systems as small contaminates can dominate the overall solution or material properties.^18^ This need is compounded when investigating cyclic BBPs as the side chains introduce another layer of complexity that impacts dynamics and rheological properties.^6,19,20^ Thus, as the complexity of the BBP topology increases, purification and production of pure rings becomes vital to their study.^7,12,19–22^

Considering the challenges associated with the synthesis and purity of cyclic polymers, an intriguing solution lies in the utilization of biologically sourced plasmid DNA (pDNA). pDNA natively exists as a double-stranded supercoiled (SC) cyclic polymer that can be relaxed or cut enzymatically to yield the open-circular (OC) or linear (L) form respectively.^23^ Interestingly, each isoform exhibits altered biological properties, such as circular DNA's increased resistance to thermal and enzymatic degradation *versus* L counterparts.^23–26^ Beyond topology, pDNA varies widely in length (2–20 kilobase pairs, kbp, 0.340 nm per bp) while remaining unimolecular (*Đ* = 1) which can greatly simplify determination of structure–property relationships.^22,24–27^ Accordingly, DNA is routinely used as a model polymer and was instrumental in developing models to describe the dynamics of entangled solutions of linear polymers.^27–34^ Leveraging the unique topological forms, unimolecular dispersity, and wealth of physical data available, DNA is an ideal polymeric backbone to generate linear and cyclic BBPs.^35–38^ However, significant challenges exist in the synthesis and functionalization of biologically derived DNA.

First, DNA must be obtained on a sufficient scale for study. Synthetic methods (*e.g.* solid phase oligonucleotide synthesis) and molecular biology methods (*e.g.* rolling circle amplification or polymerase chain reaction) remain prohibitively costly at scale and only produce linear DNA.^39–41^ Sheared genomic DNA has recently gained traction as a sustainable and inexpensive source of nucleic acids from biologic sources but only produces samples with high dispersity.^28,42–44^ Alternatively, pDNA can be obtained through simple and inexpensive fermentation with optimized protocols yielding up to 1–2 mg of pDNA per liter of culture.^45^ Unfortunately, this would require hundreds of liters to obtain a sufficient amount of pDNA for bulk mechanical study and excludes the significant purification steps required to isolate pDNA from the bacterium without shearing or introducing significant linear contaminants. To circumvent this issue, our group has recently reported a method to isolate and purify pDNA from bioreactors which can inexpensively and efficiently produce up to 1 g of unimolecular pDNA per batch.^46–48^ Access to gram scale quantities of linear and cyclic DNA provides topologically defined polymers which could be subsequently transformed into BBPs after grafting.

**Fig. 1:**
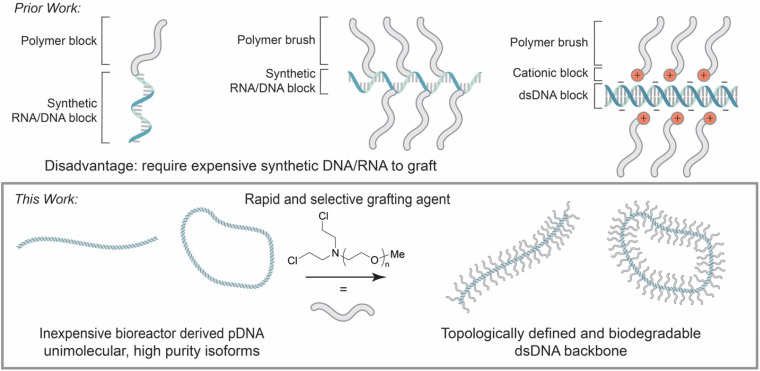
An illustration comparing the structure of previously reported DNA/RNA block copolymers and BBPs to the ones presented in this publication. The bottom section depicts the linear and cyclic DNA BBPs produced in this study which are distinct from the above work.

Another major challenge in DNA BBP synthesis is the small number of chemistries available to append functionality to native DNA. Typically, solid phase oligonucleotide synthesis is employed to add non-native functionality to DNA or RNA for subsequent functionalization with polymers or initiators.^49^ Wiel, Ng, and many others have reported unique and useful DNA and RNA containing block copolymers leveraging functionality installed through solid phase oligonucleotide synthesis (Fig. 1).^50–52^ Similarly, Matyjaszewski has reported examples of DNA and RNA backbone BBPs synthesized using a graft-from approach and ATRP (Fig. 1). Unfortunately, both cases require a pre-functionalized single stranded oligonucleotide backbone or employs alkylating agents that are selective only for RNA.^53–56^ The de Vries group demonstrated a dsDNA BBP using electrostatic attraction to graft the chains to the backbone, but the dynamic nature of this interaction is not amenable to many applications of BBPs (Fig. 1).^36^

**Fig. 2:**
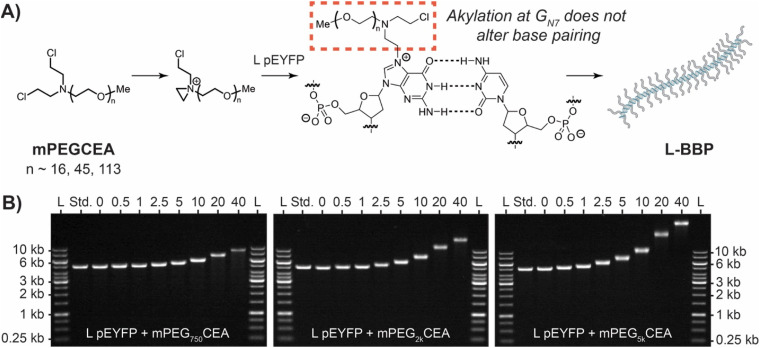
(A) Mechanism and formation of DNA BBPs using CEA. CEAs form an aziridinium intermediate that ultimately alkylates the N_7_ guanine as the major product. It does not typically react at the base pairing face preserving structure. When L pEYFP is reacted with excess mPEGCEA a linear DNA BBP (L-BBP) is formed. (B) Gel electrophoresis analysis for alkylation experiments of L pEYFP (5045 bp) with mPEGCEAs on a 0.5% agarose gel. L pEYFP was reacted with various concentrations of mPEG_750_CEA (left), mPEG_2k_CEA (middle), or mPEG_5k_CEA (right) for 1.5 h at 37 °C to yield L-BBPs. L stands for the GeneRuler 1 kb molecular weight ladder and the standard was unfunctionalized L pEYFP. The numbers at the top of each gel represent the equivalents of mPEGCEA added relative to the number of nucleotides in each sample.

As we are constrained to using biologically sourced DNA to achieve control over BBP topology, we needed to develop a graft-to or graft-from approach that will efficiently attach polymer chains without compromising the base pairing or topology. While generally considered to be relatively inert, DNA is weakly nucleophilic and strong electrophiles could serve as potential candidates to append polymers or initiators. Non-specific DNA alkylators, such as nitrosamines, epoxides, acrylates, and alkyl sulfonates have all been employed to attach dyes, pharmaceuticals, and fluorescent probes to random nucleotides.^44,57–64^ However, these reagents can degrade DNA, have low selectivity for DNA over other biomolecules, or alkylate in the base pairing face which compromises any instilled topology.^44,57–64^ A review of DNA therapeutics revealed that chloroethylamines (CEAs) are capable of more selectively reacting with dsDNA, work in biologically relevant conditions, and do not denature dsDNA. The reactivity of chloroethylamines with dsDNA has been shown to react preferentially with the external guanine N_7_ position and presented as an attractive option for attaching side chains to our DNA backbone (Fig. 2A).^57,60^

Herein, we report on the development of topologically defined linear and cyclic DNA BBPs using pUC19 (2.686 kb) and pEYFP (5.045 kb) as our backbones and mPEG functionalized with chloroethylamines (CEA) as side chains. High graft density (>60% of base pairs) is accessible through excess stoichiometric alkylator concentration. Subsequent investigation of the rheological properties of linear (L-BBP) and ring (R-BBP) BBPs compared to their unmodified counterparts revealed topology dependent mechanical properties. Finally, as the stability of DNA BBPs is important for many end use applications, we explored their enzymatic resistance to DNase I and found that PEGylation of DNA provides some protection at higher graft densities.

## Experimental

### Materials and reagents

Chloroform and toluene were purchased from Fisher Chemical. Polyethylene glycol monomethyl ether (*M*_w_ = 750, 2000, and 5000 g mol^−1^), diethanolamine, anhydrous *N*,*N*-dimethylformamide (DMF), and Amicon Ultra 0.5 and 15 mL centrifugal filter units (10 kDa MWCO) were purchased from Sigma Aldrich. Triethylamine, sodium iodide, and thionyl chloride were purchased from Oakwood Chemical. 10× Tris-Acetate-EDTA (TAE) solution and methanesulfonyl chloride were purchased from Thermo Fisher Scientific. 10× TAE was diluted with ultrapure water from a Barnstead MicroPure water system to 1× before use. Unless stated otherwise, all reagents and materials were used as supplied and/or directed by the manufacturer.

### Instrumentation

Bacterial cultures were grown in a 7 L vessel using an Applikon ez-2 control bioreactor. Anion exchange chromatography was performed using a Bio-Rad NGC chromatography system. ^1^H NMR spectra were measured using a Bruker 700 MHz spectrometer. Chemical shifts are reported in parts per million using CDCl_3_. All spectra can be found in the ESI.† Electrophoresis images were obtained using a Syngene GeneFlash BioImaging system and processed using ImageJ. All rheological measurements were taken using a TA Instruments Discovery HR-20 rheometer with a 40 mm sandblasted parallel plate geometry and solvent trap.

### Production and purification of pDNA

pDNA samples were obtained using methods previously reported by our group which couples a fed-batch fermentation process with a modified alkaline lysis method and anion exchange chromatography to access gram-scale quantities of pure pDNA solutions.^46^ Briefly, *E. coli* transformed with the target plasmid were grown in a 7 L bioreactor using a semi-defined growth media. Upon reaching a target OD_600_ of 10, a chemically defined feed medium was added at a constant rate for the duration of the culture. Upon reaching stationary phase (OD_600_ ∼ 90), the culture was chilled to 25 °C for 30 min before being chilled to 15 °C. Cells were then harvested using centrifugation and lysed using a modified alkaline lysis method to create a crude pDNA solution in which the major contaminant is lower *M*_w_ RNA. Pure samples were then obtained using anion exchange chromatography and concentrated using isopropanol precipitation. Concentration and purity of samples are measured using a Nanodrop One^C^ spectrophotometer. Quality and isoform content of samples were characterized using AGE and gel band densitometry analysis in ImageJ.

### Synthesis of mPEGOMs

Polyethylene glycol monomethyl ether (mPEG-OH) was dissolved in chloroform and cooled to 0 °C using an ice bath. Triethylamine (5 eq.) was added to the solution and allowed to stir for 10 min. Methanesulfonyl chloride (5 eq.) was then added dropwise to the reaction mixture which was then allowed to warm to room temperature and stirred for 16 h. The reaction mixture was then diluted with chloroform (0.1 g mPEG mL^−1^) and washed with 1 M HCl (1× 1 vol) followed by brine (1× 1 vol). A majority of the solvent was removed under reduced pressure to yield the crude product as orange oil. Product was precipitated through the addition of ether (10× v/v). The mixture was cooled to −20 °C overnight before the solids were collected by vacuum filtration and washed with additional cold ether to yield the product as either a tacky beige solid (mPEG_750_OMs) or white powder (mPEG_2k_OMs and mPEG_5k_OMs). Detailed procedures and NMR spectra can be found in the ESI (Fig. S1–S3).†

### Synthesis of mPEGDEA

mPEGOMs was dried under reduced pressure with heating at 60 °C for 1 h. The dried compound was then dissolved in anhydrous DMF under nitrogen. Sodium iodide was then added to the solution and allowed to stir for an additional 10 min at 60 °C under nitrogen. Diethanolamine (5 eq.) was then added dropwise under nitrogen. The reaction mixture was then heated to 100 °C and allowed to stir for 72 h under nitrogen. The reaction mixture was cooled to room temperature and the solvent was removed under reduced pressure. The resulting dark orange oil was dissolved in chloroform (0.1 g mPEG mL^−1^) and washed with saturated bicarbonate solution (1× 1 vol). A majority of the solvent was removed under reduced pressure to yield the crude product as an orange oil. Product was precipitated through the addition of ether (10× v/v). The mixture was cooled to −20 °C overnight before the solids were collected by vacuum filtration and washed with additional cold ether to yield the product as either a tacky yellow solid (mPEG_750_DEA) or beige powder (mPEG_2k_DEA and mPEG_5k_DEA). Detailed procedures and NMR spectra can be found in the ESI (Fig. S4–S6).†

### Synthesis of mPEGCEA

mPEGDEA was dissolved in thionyl chloride (40 eq.) and refluxed at 80 °C for 16 h. The reaction was diluted with toluene (1 : 10) and then the solvent was removed under reduced pressure. Excess thionyl chloride was then removed through co-evaporation with toluene (3 × 1 vol). The resulting dark orange oil was dissolved in chloroform (0.1 g mPEG mL^−1^) and washed with saturated bicarbonate solution (1× 1 vol). A majority of the solvent was removed under reduced pressure to yield the crude product as an orange oil. Product was precipitated through the addition of ether (10× v/v). The mixture was cooled to −20 °C overnight before the solids were collected by vacuum filtration and washed with additional cold ether to yield the product as either a tacky orange solid (mPEG_750_CEA) or beige powder (mPEG_2k_CEA and mPEG_5k_CEA). Detailed procedures and NMR spectra can be found in the ESI (Fig. S7–S9).†

### Synthesis of DNA BBP conjugates

A stock solution of mPEGCEA (50 mM) was made using 1× TAE and diluted as needed. Alkylator solutions were made fresh each time and used within 10 minutes of complete dissolution. pDNA solution, 1× TAE, and alkylator solutions were combined in either a 1.5 mL Eppendorf tube or a 15 mL conical tube. Samples were homogenized by gently inverting and vortexing for 3 s and then centrifuged in either a microcentrifuge (1.5 mL) or a hand-crank centrifuge (15 mL) for 15 s. Reactions were then incubated at 37 °C for 1–2 h. Success of alkylation was assessed using agarose gel electrophoresis. Final pDNA concentration was 0.1 mg mL^−1^ for small-scale reactions and 1 mg mL^−1^ for large-scale reactions. Excess unreacted mPEGCEA was removed from large-scale reactions using Amicon Ultra-15 centrifugal filter units with a molecular weight cutoff of 10 kDa. Detailed procedure can be found in the ESI.†

### DNase I activity tests

DNase I (1 U μL^−1^) was freshly diluted with 1× reaction buffer + MgCl_2_ to a final concentration of 0.001 U μL^−1^. 0.1 μg of DNA or DNA BBP was diluted with nuclease-free water (to 20 μL total volume) and 10× reaction buffer with MgCl_2_ (2 μL). Diluted DNase I (2 μL, 0.0001 U μL^−1^) was added to initiate the reaction and samples were incubated at 22 °C for 5, 10, 20, 40, or 60 minutes on a dry heat block. Reactions were stopped by adding EDTA (25 mM) to inactivate enzymes. Extent of degradation in each sample was analyzed using agarose gel electrophoresis. Percentage of higher molecular weight DNA (>1 kb or 617 kDa) was determined using gel band densitometry analysis in ImageJ.

### Agarose gel electrophoresis

Unless stated otherwise, DNA BBP conjugates were characterized using 12 cm, 0.5% agarose gels that contained 0.35 g mL^−1^ of ethidium bromide. Gels were loaded with 0.25 μg of the GeneRuler 1 kb ladder and 0.05 μg of standards and samples. The running buffer was a 40 mM tris, 20 mM sodium acetate, 2 mM EDTA, pH 8.3 solution that was purchased as a 10× stock. Gels were run at 75 V for 1–2 h and visualized using a UV transilluminator to determine completion before imaging on Syngene GeneFlash BioImaging.

## Results and discussion

To develop a graft-to agent, bis-CEA was appended to the end of polyethylene glycol monomethyl ether to yield mPEGCEA (Fig. 2A, see ESI† for details). As the pDNA solutions from the bioreactor contain a variable mixture of SC and OC DNA, a standard protocol for DNA BBP production and characterization was first established using linearized pDNA (L-DNA). A freshly made solution of mPEG_2k_CEA was combined with L pEYFP at 37 °C for 1.5 h in 1× TAE buffer, pH 8.3. mPEG_2k_CEA was added relative to μmole of nucleotides in DNA (0.5–40 eq.) and alkylation was analyzed by agarose gel electrophoresis (Fig. 2B). As increasing equivalence of mPEG_2k_CEA were added, a single band of increasing apparent molecular weight was observed, suggesting successful conjugation and production of linear DNA BBPs (L-BBP) (Fig. 2B). UV-Vis absorbance at 260 nm before and after grafting confirmed no loss in double stranded nature (Table S1†). This was then repeated with mPEG_750_CEA and mPEG_5k_CEA which yielded similar results. As expected, L-BBP made with the shortest mPEGCEA produced the smallest shifts while L-BBPs made with the longest mPEGCEA produced the greatest shift in apparent molecular weight (Fig. 2B). Graft density was estimated using the retention factor (*R*_f_) of the L-BBP relative to the *M*_w_ of the ladder and the standard run with each sample (Table 1). Large decreases in *R*_f_ indicate relatively high levels of grafting, with over 60% estimated grafting density observed for each sample at 40 eq. A larger molecular weight ladder was used to confirm that the *R*_f_ estimations for the largest BBPs could be extrapolated from the smaller ladder (Fig. S1†). We note that a large excess of reagent is necessary to achieve reasonable graft densities likely due to both the inefficiency of the graft-to approach, and the hydrolysis of CEAs in aqueous media (*t*_1/2_ ∼ 1 h).^1,60,65^ Additionally, the grafting of PEG to DNA likely shields charge therefore, migration of DNA BBP conjugates is not due to increase of size alone. However, until better methods are established, this approach provides a quantitative method for comparing DNA BBPs.

**Table 1:** Results of linear DNA BBP graft-to synthesis

Equivalence	Apparent *M*_w_ a (kDa)	Graft density^b^ (%)
L pEYFP + mPEG_750_CEA		
0	3158	0.0
0.5	3272	2.6
1	3310	3.5
2.5	3511	8.1
5	3680	12.0
10	4090	21.5
20	4994	42.3
40	6098	67.8

L pEYFP + mPEG_2k_CEA		
0	3187	0.0
0.5	3350	1.5
1	3407	2.0
2.5	3778	5.5
5	4232	9.7
10	5310	19.7
20	7846	43.2
40	10 594	68.7

L pEYFP + mPEG_5k_CEA		
0	3216	0.0
0.5	3441	0.9
1	3619	1.6
2.5	4164	3.7
5	4994	6.9
10	6775	13.7
20	12 614	36.3
40	19 511	62.9

a Apparent *M*_w_ calculated by extrapolation based on the 1 kb DNA ladder reference.

b Grafting density calculated based on the difference in extrapolated *M*_w_ values for each DNA BBP conjugate.

**Fig. 3:**
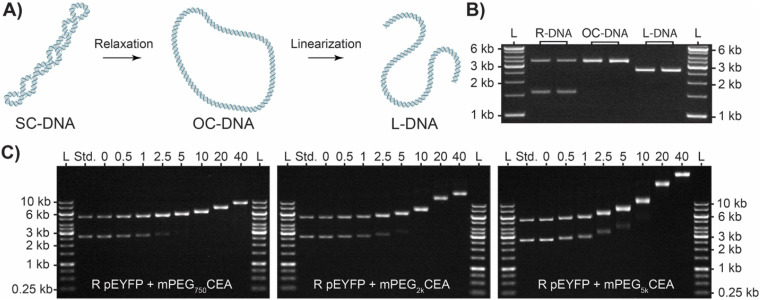
(A) Illustration depicting the conversion of supercoiled pDNA (SC-DNA) to open circle (OC-DNA) and linear (L-DNA) isoforms. (B) Gel electrophoresis analysis for the digestion of pUC19 (2.686 kb) with either Nt. BspQI or EcoRI to show the differences in migration for OC and L isoforms on a 1% agarose gel. Lane 1: GeneRuler 1 kb molecular weight ladder, 2: ring pUC19 obtained from our bioreactor containing SC and OC DNA, lanes 3 and 4: pUC19 after treatment with Nt. BspQI producing 100% OC-DNA, lanes 5 and 6: pUC19 after treatment with EcoRI producing 100% L-DNA. (C) Electrophoresis analysis for alkylation experiments of R pEYFP (5.045 kb) with mPEGCEAs on a 0.5% agarose gel. R pEYFP was reacted with various concentrations of mPEG_750_CEA (left), mPEG_2k_CEA (middle), or mPEG_5k_CEA (right) for 1.5 h at 37 °C to yield R-BBPs. L stands for the GeneRuler 1 kb molecular weight ladder and the standard was unfunctionalized R pEYFP. The numbers at the top of each gel represent the equivalents of mPEGCEA added relative to the number of nucleotides in each sample.

Having established a graft-to method for production and characterization of L-BBPs using mPEGCEAs we next sought to produce their cyclic analogs (R-BBP). pDNA obtained from our bioreactor process contains a variable mixture of SC/OC DNA (Ring DNA or R-DNA) resulting from chemical and mechanical nicking during alkaline lysis and purification process (Fig. 3A). It should be noted that SC, OC, and L isoforms of DNA are easily distinguishable from each other *via* their relative *R*_f_ as they have unique electromotive forces based on their topology (Fig. 3B). When the R-DNA solution was mixed with each of the mPEGCEAs, a similar decrease in *R*_f_ was observed and used to estimate graft density (Fig. 3C and Table 2). Interestingly, as graft density increases, chemical nicking and subsequent relaxation of SC to OC DNA increases until only OC-BBP is observed. We hypothesize that this is due to the increased strain energy SC DNA experiences during alkylation and chemical induced nicking of the phosphate backbone.^57–60,66,67^ As only the disappearance of the SC-BBP is observed, we can deduce no linearization and contamination from L-BBPs is occurring.

**Table 2:** Results of ring DNA BBP graft-to synthesis

OC			SC	
Equivalence	Apparent *M*_w_ a (kDa)	Graft density^b^ (%)	Apparent *M*_w_ a (kDa)	Graft density^b^ (%)
R pEYFP + mPEG_750_CEA				
0	3627	0.0	1667	0.0
0.5	3663	0.8	1717	1.2
1	3735	2.5	1751	1.9
2.5	3847	5.1	1876	4.8
5	4081	10.5		
10	4503	20.2		
20	5323	39.1		
40	6418	64.3		

R pEYFP + mPEG_2k_CEA				
0	3589	0.0	1639	0.0
0.5	3743	1.4	1696	0.3
1	3828	2.2	1754	0.9
2.5	4026	4.1	1881	2.1
5	4430	7.8		
10	5332	16.2		
20	8691	47.3		
40	10 519	64.3		

R pEYFP + mPEG_5k_CEA				
0	3796	0.0	1710	0.0
0.5	4102	1.2	1852	0.5
1	4295	1.9	2139	1.7
2.5	5036	4.8	2395	2.6
5	5979	8.4	2947	4.8
10	8271	17.3		
20	16 093	47.5		
40	23 165	74.8		

a Apparent *M*_w_ calculated by extrapolation based on the 1 kb DNA ladder reference.

b Grafting density calculated based on the difference in extrapolated *M*_w_ values for each DNA BBP conjugate.

**Fig. 4:**
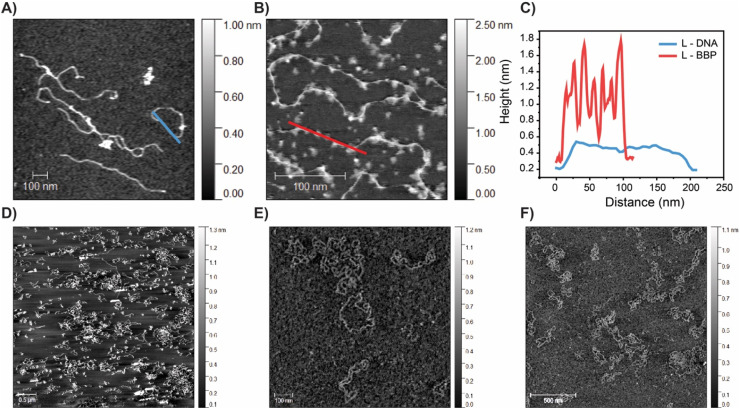
AFM micrographs of DNA and DNA BBPs. (A) Dry AFM of L pUC19. Scale bar is 100 nm. Height measurement is made along blue line. (B) Dry AFM of 10% grafted mPEG_2k_CEA with L pUC19. Scale bar is 100 nm. Height measurement is made along red line. (C) Height comparison of L pUC19 and L DNA-BBP as shown in A and B. The increased height likely arises from the grafting of mPEG. (D). Dry AFM of R pUC19. Scale bar is 500 nm. (E) Liquid phase AFM of 30% grafted mPEG_750_CEA with R pUC19. Scale bar is 100 nm. (F) Larger image of sample E, demonstrating retention of circularity of polymers after alkylation. Measurements cannot be made between dry/wet sample.

To further confirm the topology and successful formation of the different DNA BBPs, select samples were dry imaged using atomic force microscopy (AFM). First, R and L pUC19 were imaged to serve as a point of comparison (Fig. 4A and D). We next performed dry imaging of L pUC19 grafted with mPEG_2k_CEA (∼10% grafting) (Fig. 4B). This sample revealed a pearl necklace appearance where the areas of high graft density are observed as “pearls” and unreacted polymer can be observed in the background. When trying to dry image sample with a higher grafting density, no BBP could be observed. Typically, when imaging DNA, freshly cleaved mica is pretreated with a NiCl_2_ solution to ensure electrostatic adherence of the sample to the substrate before subsequent washing, drying, and imaging. We hypothesize that the steric shielding of PEG brushes was blocking the backbone from interacting with the mica surface.^68–73^ To image a sample with higher graft density, direct liquid imaging was used. R pUC19 with a higher graft density (mPEG_750_CEA, ∼30% grafting) was successfully imaged to confirm that no linearization was observed (Fig. 4D, E and F). Based on our combined imaging and electrophoresis results, we can confirm the production and purity of both linear and cyclic DNA BBPs. Imaging the DNA BBPs grafted with larger *M*_w_ PEG at higher densities has proven challenging and is the focus of future studies.

**Fig. 5:**
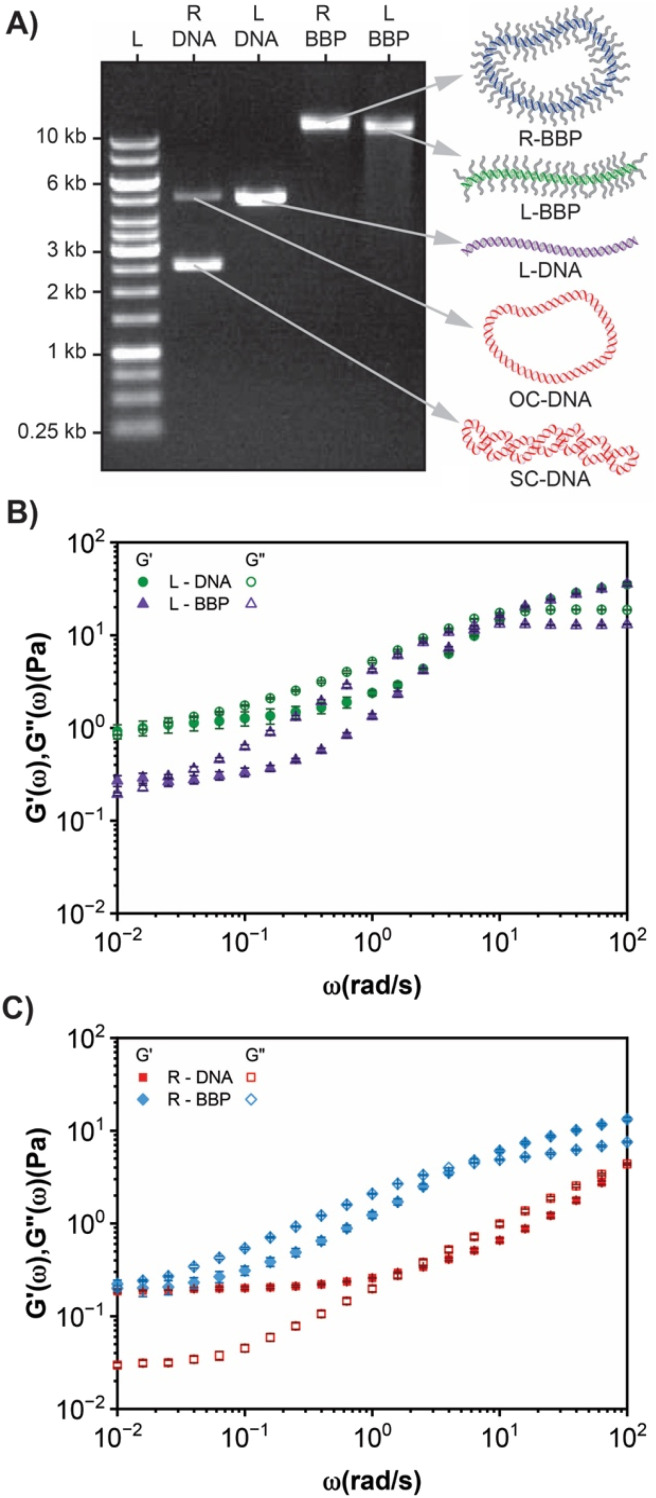
(A) Gel electrophoresis analysis of large-scale alkylation of L (L-DNA) and R (R-DNA) pEYFP on a 0.5% agarose gel. Lane 1: GeneRuler 1 kb molecular weight ladder, lane 2: R pEYFP, lane 3: L pEYFP, lane 4: ring DNA BBP (R-BBP) with a graft density of 42.2% and lane 5: linear DNA BBP (L-BBP) with a graft density of 40.1%. (B) Bulk linear oscillatory rheology of L-DNA (green circles) and L-BBP (purple triangles) at 10 mg mL^−1^. (C) Bulk linear oscillatory rheology of R-DNA (red squares) and R-BBP (blue diamonds) at 10 mg mL^−1^. Linear viscoelastic moduli, *G*′ (*ω*)(storage modulus, closed symbols) and *G*′′ (*ω*)(loss modulus, open symbols) *versus* angular frequency, *ω*. Data shown are an average over 3 independent measurements and the error bars represent the standard error. All measurements were taken on a TA instruments discovery HR-20 rheometer with parallel plate geometry.

To determine the mechanical properties of DNA BBP solutions it was necessary to scale up the synthesis to obtain enough sample for measurement *via* shear rheology. This was accomplished using pEYFP (5.045 kb) and mPEG_2k_CEA. R pEYFP (10.6 mg) and L pEYFP (9.1 mg) were each alkylated with 10 eq. of mPEG_2k_CEA (Fig. 5A). On a larger scale the functionalization performed similarly to the small-scale preparation however, the concentration of DNA in these reactions was increased from 0.1 mg mL^−1^ to 1 mg mL^−1^ to reduce the required volume. Under these conditions, a higher graft density is achieved at a given stoichiometric equivalence (Fig. S2†). To prevent interference in subsequent rheological and enzymatic studies it was necessary to purify the large excess of grafting agent from the samples. Typically, precipitation of DNA in ethanol or isopropanol can remove soluble chemical contaminants, but the DNA BBPs remained in solution thus requiring an alternative method of purification. Interestingly, the increased solubility of DNA BBPs in organic solvents could greatly expand their potential applications. The DNA BBPs were ultimately purified and concentrated *via* centrifugal spin filters to yield solutions with a concentration of ∼10 mg mL^−1^ which is above the overlap concentration of ∼3 mg mL^−1^ for pDNA of this size.^46^ The graft density of the resulting L-BBP and R-BBP was 40.1% and 42.2%, respectively. For the larger pEYFP plasmid and the resulting BBPs, the observed *R*_f_ of the linear and OC isoforms are closer, but still distinguishable confirming they are indeed the OC and L isoforms (Fig. 5A).

Due to the ability to make large amounts of sample, we investigated the mechanical properties of the DNA BBP samples using shear rheology at a concentration of 10 mg mL^−1^. The integrity of samples before and after rheological analysis was characterized using AGE to ensure that sample aging or shear induced linearization did not influence the observed viscoelastic properties (Fig. S3†). Due to the extreme length of DNA, a physical gel is formed at relatively low sample concentration (<1 mg mL^−1^).^29,74^ We first sought to explore the dynamics of our linear systems which exhibit predicted behavior of entangled polymers and BBPs (Fig. 5B).^1,3,29,74–76^ In our L-DNA sample, the system is predominantly in the transition zone where *G*′ < *G*′′ and scaling of *G*′(*ω*) ∼ *ω*^0.9^ and *G*′′(*ω*) ∼ *ω*^0.6^ is observed. Conversely, in the L-BBP sample we see a decrease in the viscoelastic moduli at low frequencies (*ω* < 10^0^ rad s^−1^) compared to the unmodified L-DNA. The steric repulsion of the grafted side chains hinders chain packing and decreases interaction between polymer chains, acting as a diluting agent, which manifests as a decrease in the observed viscoelastic moduli.^1,3,65,75^ This is suggested by the steeper scaling of *G*′(*ω*) ∼ *ω*^1.2^ and *G*′′(*ω*) ∼ *ω*^0.8^ observed in the L-BBP.^76^ Interestingly, at high frequencies *G*′(*ω*) coalesces for both samples as both samples crossover into an elastic dominant regime. Typically, the bottlebrush backbone becomes more rigid due to the steric repulsion of the side chains, while the dilution effect decreases entanglements, lowering *G*′(*ω*) across all frequencies.^1,3,65,75^ However, as the DNA backbone is already relatively stiff (persistence length = 50 nm), grafting of relatively small PEG_2k_ will likely have little effect on entanglement density at higher frequencies. When observing the R-DNA and R-BBP, an elastic plateau modulus is observed in the R-DNA sample at lower frequencies where *G*′ > *G*′′ and *G*′(*ω*) ∼ *ω*^0.1^ and *G*′′(*ω*) ∼ *ω*^0.7^ (Fig. 5C). At higher frequencies, we see a crossover (*ω*_c_ = 1.6 rad s^−1^) into a viscous dominated regime where *G*′ < *G*′′ and scaling of *G*′(*ω*) ∼ *G*′′(*ω*) ∼ *ω*^0.7^ is observed_._ The plateau zone is not observed in the R-BBP sample which is predominantly in the transition zone where *G*′(*ω*) ∼ *ω*^0.7^ and *G*′′(*ω*) ∼ *ω*^0.6^ before crossing over (*ω*_e_ = 6.3 rad s^−1^) to an elastic dominant regime. We note that this may not be a “true” change in the plateau modulus, but intrinsic to the bottle brushes polymers having a longer relaxation time. We hypothesize that there are two contributing causes to this phenomenon: (1) transition from a SC/OC mixture to exclusively OC, and (2) increased interaction of R-BBPs *via* their sidechains. Of the limited data reported for supercoiled and open circle solutions, blends exhibit dynamics like those of entangled linear polymers which is supported by the elastic plateau modulus observed in our ring pEYFP frequency sweeps.^77^ The exact fraction of supercoiled species is not quantified and also may introduce a higher entanglement density. While the impact of the SC : OC ratio has not been explored in detail, data out of our lab has shown that an increase in the OC content of the sample enhances the observed elastic plateau modulus (Fig. S4†). While the dynamics of ring polymers are still not fully understood, their ability to entangle and undergo “modified” reptation is an area of extreme debate and active investigation within the polymer's community.^18,24,46,74,77–80^ At this point, it is unclear whether change in OC : SC ratio or addition of brushes is driving the observed rheological differences. To truly investigate the properties of cyclic BBPs we will need to study solutions of pure OC and SC BPPs with much greater graft densities and brush arm lengths and thus is the subject of future studies.

**Fig. 6:**
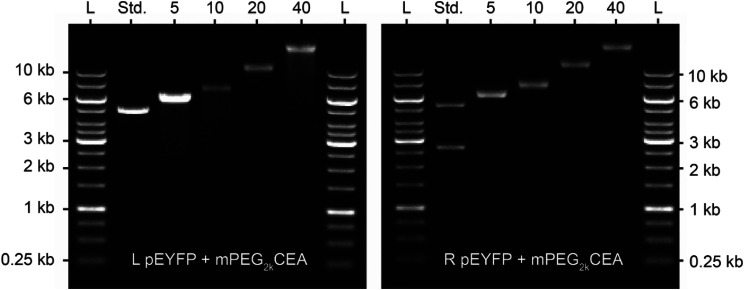
Gel electrophoresis analysis of L-BBPs (left) and R-BBPs (right) used in DNase I activity tests on a 0.5% agarose gel. L and R pEYFP were reacted with 5, 10, 20 or 40 equivalents of mPEG_2k_CEA. L-BBPs had graft densities of 10.1, 19.7, 44.4, and 78.9% while R-BBPs had graft densities of 9.2, 19.5, 47.9, and 79.8%. L stands for the GeneRuler 1 kb molecular weight ladder and the standard was unfunctionalized L pEYFP for L-BBPs and unfunctionalized R pEYFP for R-BBPs. The numbers at the top of each gel represent the equivalents of mPEG_2k_CEA added relative to the number of nucleotides in each sample.

**Fig. 7:**
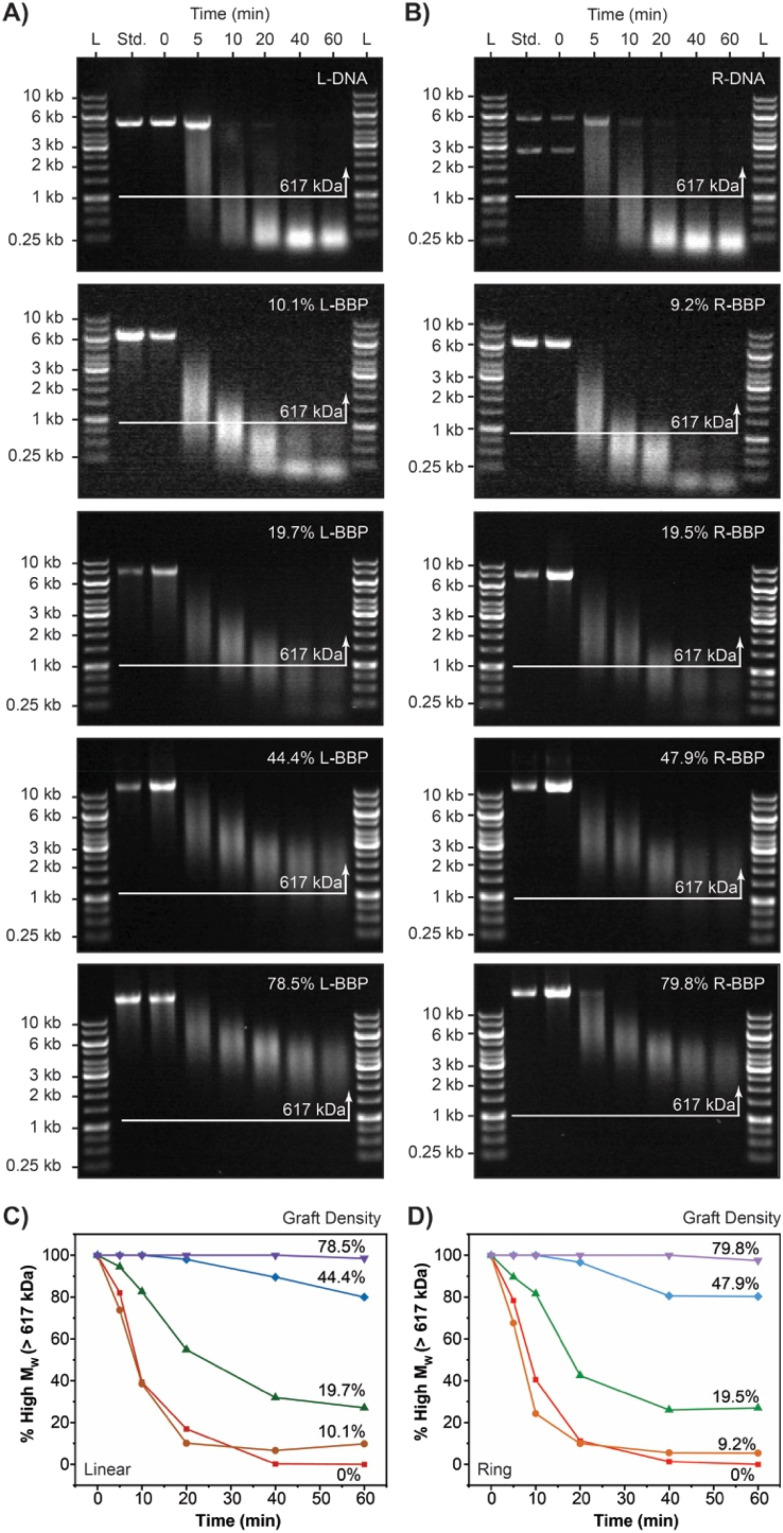
Enzymatic stability tests of DNA BBPs using pEYFP and DNase I. (A) Gel electrophoresis analysis of degradation of L-DNA and L-BBP conjugates on a 0.5% agarose gel. L-BBPs had graft densities of 10.1, 19.7, 44.4, and 78.5%. (B) Gel electrophoresis analysis of R-DNA and R-BBP conjugates on a 0.5% agarose gel. R-BBPs had graft densities of 9.2, 19.5, 47.9, and 79.8%. For (A) and (B), L stands for the GeneRuler 1 kb molecular weight ladder and standards were samples of corresponding DNA or DNA BBP before exposure to reaction buffer or enzyme. The numbers at the top represent the incubation time for each digest at 22 °C. (C) Percentage of higher molecular weight DNA (>1 kb or 617 kDa) of each L-BBP compared to unmodified L-DNA in the presence of DNase I as a function of time. (D) Percentage of higher molecular weight DNA (>1 kb or 617 kDa) of each R-BBP compared to unmodified R-DNA in the presence of DNase I as a function of time. For (C) and (D), the percentage of higher molecular weight DNA was determined by gel band densitometry analysis.

Next, due to the increased steric encumbrance of the DNA BBP, we investigated their degradation in the presence of DNAase I. Synthetic BBPs typically have all carbon backbones which suffer from limited ability to be degraded which complicates biological applications.^15,81,82^ On the contrary, DNA constructs, DNA origami, and DNA nanomachines are all rapidly degraded by endogenous DNase hindering their use. We hoped that PEGylation of DNA would increase the lifetime a of the DNA BBP, while retaining biodegradability on a longer timescale. To test this, we prepared L-BBP and R-BBP samples with varying graft densities and exposed them to a non-specific endonuclease: DNAase I. 50 μg of L and R pEYFP was alkylated with mPEG_2k_CEA at 5, 10, 20, and 40 eq. to achieve DNA BBPs with target graft densities of ∼10, 20, 40 and 70% (Fig. 6). Purified DNA and DNA BBPs were then incubated at 22 °C in the presence of DNase I (0.0001 U μL^−1^) for either 5, 10, 20, 40, or 60 minutes (Fig. 7A and B). Extent of degradation was assessed by quantifying the percent of higher molecular weight DNA observed at the end of the incubation period (Fig. 7C and D). We defined our higher molecular weight cut off as 1 kb or 617 kDa, similar to previous studies.^83^ Unsurprisingly DNA BBPs with low graft densities (<20%) exhibited little to no resistance towards enzymatic activity and were degraded almost as quickly as the unmodified DNA samples. In both linear and ring systems, almost no higher molecular weight DNA was observed beyond 10 minutes. At higher graft densities, our BBPs exhibited an increased resistance towards degradation which is supported by the observation of higher molecular weight DNA in these samples even after 300 minutes (Fig. S4†). Complete degradation of DNA BBPs with higher graft densities was not observed under the time scales assessed. These results highlight a major advancement for the use of oligonucleotides in the context of drug delivery and DNA origami as pre-mature degradation is a major obstacle in the field. This strategy is similar to PEGylation of proteins and can potentially be used to increase the half-life of DNA constructs like DNA Origami in blood.^38,83,84^

## Conclusions

In summary, we have developed a methodology for synthesizing and characterizing both linear and cyclic DNA BBPs. We estimated the graft density of chains attached to these conjugates using *R*_f_. Atomic force microscopy (AFM) provided direct observational evidence of successful conjugation of mPEG to DNA backbones and preservation of topology. By employing spin filtration, we efficiently purified the DNA–PEG conjugates and explored their unique rheological properties. Finally, we showed that at high graft densities, DNA BBPs can partially resist the action of DNase. Expanding the synthetic toolbox and refining the purification methods for DNA adducts under mild conditions will enable the exploration of their properties and applications in the fields of biomedical research and drug delivery. We anticipate that this orthogonal chemistry approach can be extended to other DNA conjugates for generating various nanostructures and investigating the bulk properties of these materials.

## Data availability

The data supporting this article have been included as part of the ESI.†

## Conflicts of interest

The authors declare no conflicts of interest.

## Supplementary Material

PY-016-D5PY00082C-s001
