# Supplementary material for: Generation of topologically defined linear and cyclic DNA bottle brush polymers via a graft-to approach
Source: Polym Chem. 2025 May 1;16(22):2646–58. doi: 10.1039/d5py00082c (PMC12070894; doi:10.1039/d5py00082c)
Supplement: PY-016-D5PY00082C-s001 [file PY-016-D5PY00082C-s001.pdf]

Supplementary Information (SI) for Polymer Chemistry.
This journal is © The Royal Society of Chemistry 2025

Supporting Information

Generation of Topologically Defined Linear and Cyclic DNA Bottle Brush Polymers via a Graft-to Approach

Nicholas G. Pierini,[a]† Wynter A. Paiva,[a]† Owen C. Durant,[a] Aubrianna M. Dobbins,[a] Ben B. Wheeler,[b] Matthew E. Currier,[a] James Vesenka,[b] Nathan J. Oldenhuis[a]*

Author affiliations: [a] Department of Chemistry, College of Engineering and Physical Science, University of New Hampshire, 23 Academic Way, Parsons Hall, Durham, NH 03824

[b] Department of Chemistry and Physics, University of New England, 11 Hills Beach Road, Biddeford, ME 04005

* Corresponding author

† **Equal Contribution**

Contents

1. General information 3
2. Materials 3
3. 3. Methods 4
   - 3.1. General synthesis of mPEG derivatives 4
   - 3.1.1. Synthesis of $mPEG_{750}OMs$ **(S1)** 4
   - 3.1.2. Synthesis of $mPEG_{750}DEA$ **(S2)** 5
   - 3.1.3. Synthesis of $mPEG_{750}CEA$ **(S3)** 6
   - 3.1.4. Synthesis of $mPEG_{2k}OMs$ **(S4)** 6
   - 3.1.5. Synthesis of $mPEG_{2k}DEA$ **(S5)** 7
   - 3.1.6. Synthesis of $mPEG_{2k}CEA$ **(S6)** 8
   - 3.1.7. Synthesis of $mPEG_{5k}OMs$ **(S7)** 9
   - 3.1.8. Synthesis of $mPEG_{5k}DEA$ **(S8)** 10
   - 3.1.9. Synthesis of $mPEG_{5k}CEA$ **(S9)** 10
   - 3.2. Synthesis of DNA PEG conjugates 11
   - 3.3. Agarose gel electrophoresis 12
   - 3.4. UV-Vis Absorbance measurements before and after alkylation 12
   - 3.5. Small-scale relaxation of pUC19 13
   - 3.6. Small-scale linearization of pUC19 13
   - 3.7. Large-scale linearization of pEYFP 14
   - 3.8. DNA mica deposition 14
   - 3.9. AFM imaging 14
   - 3.10. DNase I Activity Test 15
4. Supporting figures 14
   - 4.1. NMR spectra 14
   - 4.2. Agarose gels 24
   - 4.3. Bulk oscillatory rheology 26

1. General information

pDNA was isolated from DH5α cells transformed with the plasmid pUCP20T-eYFP (5045 bp). Cultures were grown in a 3L or 7L bioreactor using a fed-batch fermentation process and harvested via centrifugation using a Thermo Scientific Sorvall Lynx 4000 Centrifuge fitted with the F10-4x1000 LEX rotor. Cells were lysed using alkaline lysis method and purified using anion exchange chromatography via FPLC using a BioRad NGC Chromatography System. Homogenized pure DNA samples were used without modification or were digested with a sequence specific restriction enzyme to yield 100% linear DNA. DNA concentrations were determined using a Thermo Scientific Nanodrop One^c. Plasmid quality, purity, identity, and isoform content were analyzed using agarose gel electrophoresis. Electrophoresis images were worked up and quantified using gel band densitometry analysis in ImageJ. All reagents were used as supplied unless stated otherwise. All buffers were made in the lab using a Barnstead MicroPure Water System.

mPEG side chains were synthesized in the lab using commercially available reagents and solvents. All reagents and solvents were used as supplied unless stated otherwise. Successful synthesis and functionalization of mPEG derivatives was confirmed through proton nuclear magnetic resonance (^{1}H NMR) spectroscopy. All ^{1}H NMR spectra were recorded on a Bruker 700 MHz NMR spectrometer. Chemical shifts are reported in parts per million (ppm) and splitting patterns are assigned as s (singlet), d (doublet), t (triplet), q (quartet), m (multiplet), or br (broad). Couplings constants (J) are reported in Hz and MestReNova v15.1.0-38027 software was used to analyze all spectra. Methylene peaks for internal mPEG chain was observed at 3.65 ppm. Water in NMR solvent ($CDCl_3$) was observed at 1.70 – 1.77 ppm.

2. Materials

Sigma Aldrich: polyethylene glycol monomethyl ether 5000 (M_w: 5000 g/mol, $mPEG_{5K}$), polyethylene glycol monomethyl ether 2000 (M_w: 2000 g/mol, $mPEG_{2K}$), polyethylene glycol monomethyl ether 750 (M_w: 750 g/mol, $mPEG_{750}$), diethanolamine (DEA, for synthesis), anhydrous dimethylformamide (DMF, ACS grade). Oakwood: Sodium Iodide (NaI, ACS grade), triethylamine (TEA, reagent), thionyl chloride (>99%), HEPES (99%), Nickel (II) Chloride hexahydrate (99%). Cambridge Isotopes: dimethyl sulfoxide-d6 (DMSO-d6 (99.8%), chloroform-

d (CDCl3, 99.8%). Fisher Scientific: Methanesulfonyl chloride (MsCl, 98%), diethyl ether (ACS Grade), anhydrous sodium sulfate (ACS grade), sodium chloride (ACS grade), Agarose (TopVision), 6x DNA gel loading dye, GeneRuler 1 kb molecular weight ladder, 10X Tris-Acetate-EDTA Buffer Solution (TAE buffer). Thermo Fisher Scientific: EcoRI (10 U/µL) New England Biolabs: Nt. BspQI (10 U/µL). Ted Pella: 10 mm diameter mica (Ted Pella, 50) Oxford Instruments: AC240TSA-R3 cantilever.

3. Methods

3.1. General synthesis of mPEG derivatives

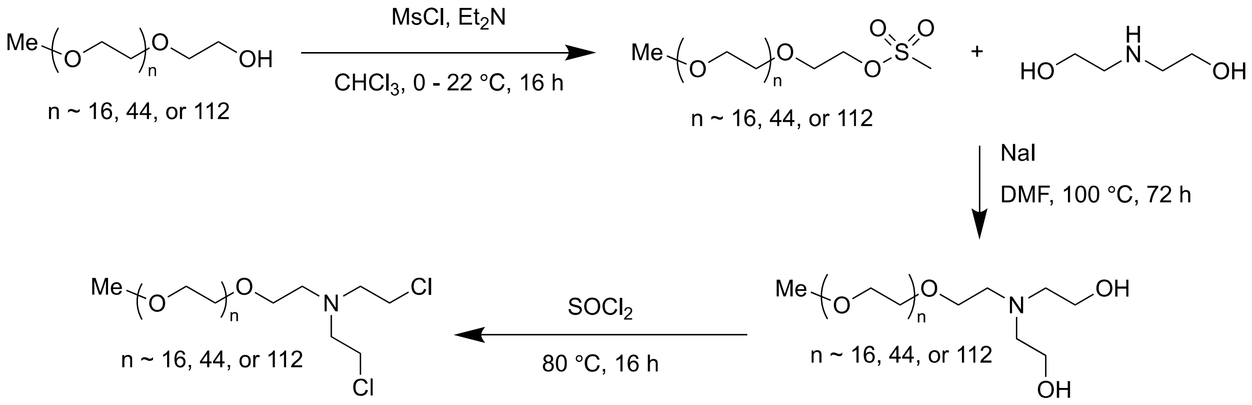

*3.1.1. Synthesis of polyethylene glycol monomethyl ether 750 mesylate (mPEG$_{750}$OMs) **(S1)***

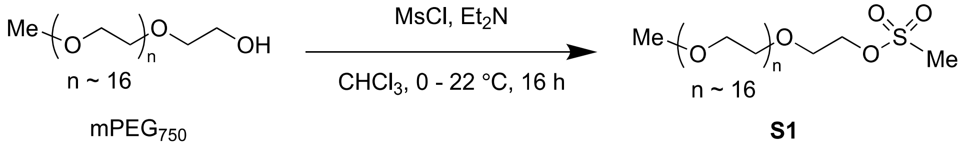

To a 100 mL round bottom flask equipped with a stir bar, monomethyl ether polyethylene glycol (mPEG$_{750}$, M_W = 750 g/mol, 5.2 g, 6.93 mmol, 1 eq.) was added and dissolved in chloroform ($CHCl_3$, 25 mL). Triethylamine (Et_2N, 3.5866 g, 35.4 mmol, 5.1 eq.) was then added to the solution. The reaction was cooled to 0 °C using an ice bath and allowed to stir for 10 min. Methanesufonyl chloride (MsCl, 4.1188 g, 35.9 mmol, 5.2 eq.) was added dropwise to the vessel. The reaction was allowed to warm to room temperature and stirred for 16 h. The reaction mixture was transferred to a separatory funnel and diluted with chloroform (25 mL) before being washed with 1 M HCl (1X 60 mL). The acidic aqueous layer was back extracted with chloroform (1X 60 mL) and the combined organic layers were washed with brine (1X 110 mL). The aqueous layer

was back extracted with chloroform (1X 110 mL) and the combined organic layers were dried over sodium sulfate and filtered. A majority of the solvent was then removed under reduced pressure to yield the crude product as a dark orange syrup. The crude product was precipitated by the addition of cold ether (10X v/v). The precipitate slurry was incubated at – 20 °C for 16. The pure product was isolated using vacuum filtration and washed with additional cold ether. Product was immediately transferred to a 100 mL round bottom flask and placed under vacuum to dry to completeness yielding the compound **S1** as a tacky beige solid (3.57 g, 63% yield, 100% functionalization).

^{1}H NMR (700 MHz, $CDCl_3$) δ: 4.39 – 4.37 (m, 2H), 3.65 (br, 82H), 3.38 (s, 3H), 3.08 (s, 3H).

*3.1.2. Synthesis of polyethylene glycol monomethyl ether 750 diethanolamine (mPEG$_{750}$DEA) (**S2**)*

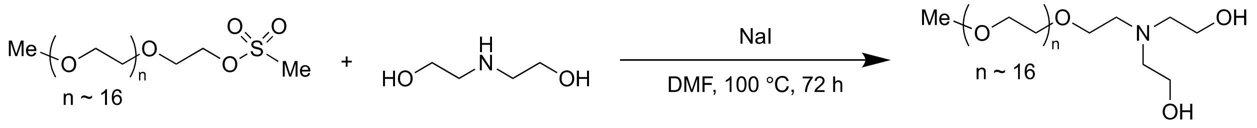

S1 **S2**

Compound **S1** (3.57 g, 4.39 mmol, 1 eq.) was dried under reduced pressure with heating at 60 °C for 1 h in a 100 mL round bottom flask. Once dried, **S1** was cooled to room temperature and dissolved in anhydrous DMF (20 mL) under nitrogen. Sodium iodide (0.6942 g, 4.6 mmol, 1.1 eq.) was added to the vessel and the reaction mixture was heated to 60 °C and allowed to stir for 10 minutes under nitrogen producing a dark red solution. Diethanolamine (2.4992 g, 22.8 mmol, 5.4 eq.) was added dropwise to the reaction vessel under nitrogen. The resulting dark yellow reaction mixture was heated to 100 °C and allowed to stir under nitrogen for 72 h. The reaction mixture was cooled to room temperature and the solvent was removed under reduced pressure to yield the crude product as a dark red syrup. The crude product was dissolved in chloroform (50 mL) and washed with a saturated bicarbonate solution (3X 60 mL). The aqueous solution was then back extracted with chloroform and the combined organic layers were dried over sodium sulfate and filtered. A majority of the solvent was then removed under reduced pressure to yield the crude product as a dark red syrup. The crude product was precipitated by the addition of cold ether (10X v/v). The precipitate slurry was incubated at – 20 °C for 16. The pure product was isolated using vacuum filtration and washed with additional cold ether. Product was immediately transferred to

a vial and placed under vacuum to dry to completeness yielding the compound **S2** as a tacky orange solid (2.31 g, 64% yield, 100% functionalization).

^{1}H NMR (700 MHz, $CDCl_3$) δ: 3.65 (br, 84H), 3.38 (s, 3H), 2.73 (t, 2H, *J* = 7.0 Hz), 2.67 (t, 4H, *J* = 7.0 Hz).

*3.1.3. Synthesis of polyethylene glycol monomethyl ether 750 chloroethylamine ($mPEG_{750}CEA$) (**S3**)*

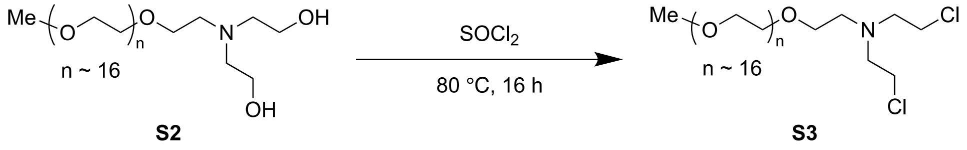

In a 50 mL round bottom flask equipped with a stir bar, compound **S2** (2.31 g, 2.8 mmol, 1 eq.) was dissolved in thionyl chloride (9 mL, 14.76 g, 124.1 mmol, 44 eq.). The resulting yellow reaction mixture was the refluxed at 80 °C for 16 h. Upon cooling to room temperature, the reaction was diluted with toluene (20 mL) and the solvent was removed under reduced pressure. Excess thionyl chloride was removed through co-evaporation with toluene (3X 20 mL) to yield the crude product was a dark orange syrup. The crude product was dissolved in chloroform (50 mL) and washed with a saturated bicarbonate solution (3X 60 mL). The aqueous solution was then back extracted with chloroform and the combined organic layers were dried over sodium sulfate and filtered. A majority of the solvent was then removed under reduced pressure to yield the crude product as a dark red syrup. The crude product was precipitated by the addition of cold ether (10x v/v). The precipitate slurry was incubated at – 20 °C for 16. The pure product was isolated using vacuum filtration and washed with additional cold ether. Product was immediately transferred to a vial and placed under vacuum to dry to completeness yielding the compound **S3** a tacky yellow solid (1.19 g, 50%, 96% functionalization).

^{1}H NMR (700 MHz, $CDCl_3$) δ: 3.65 (br, 90H), 3.38 (s, 3H), 2.95 (t, 4H, J = 7 Hz), 2.82 (t, 2H).

*3.1.4. Synthesis of polyethylene glycol monomethyl ether 2000 mesylate ($mPEG_{2K}OMs$) (**S4**)*

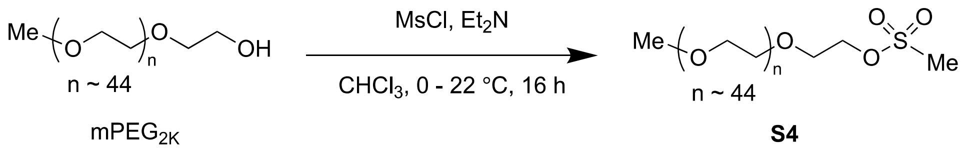

To a 100 mL round bottom flask equipped with a stir bar, monomethyl ether polyethylene glycol (mPEG$_{2K}$, M_W = 2000 g/mol, 5.0106 g, 2.51 mmol, 1 eq.) was added and dissolved in chloroform ($CHCl_3$, 25 mL). Triethylamine (Et_2N, 3.5866 g, 35.4 mmol, 5.1 eq.) was then added to the solution. The reaction was cooled to 0 °C using an ice bath and allowed to stir for 10 min. Methanesufonyl chloride (MsCl, 1.48 g, 12.9 mmol, 5.2 eq.) was added dropwise to the vessel. The reaction was allowed to warm to room temperature and stirred for 16 h. The reaction mixture was transferred to a separatory funnel and diluted with chloroform (25 mL) before being washed with 1 M HCl (1X 60 mL). The acidic aqueous layer was back extracted with chloroform (1X 60 mL) and the combined organic layers were washed with brine (1X 110 mL). The aqueous layer was back extracted with chloroform (1X 110 mL) and the combined organic layers were dried over sodium sulfate and filtered. A majority of the solvent was then removed under reduced pressure to yield the crude product as a dark orange syrup. The crude product was precipitated by the addition of cold ether (10X v/v). The precipitate slurry was incubated at – 20 °C for 16. The pure product was isolated using vacuum filtration and washed with additional cold ether. Product was immediately transferred to a 100 mL round bottom flask and placed under vacuum to dry to completeness yielding the compound **S1** as a tacky orange solid (3.57 g, 63% yield, 100% functionalization).

^{1}H NMR (700 MHz, $CDCl_3$) δ: 4.39 – 4.37 (m, 2H), 3.65 (br, 192H), 3.38 (s, 3H), 3.09 (s, 3H)

*3.1.5. Synthesis of polyethylene glycol monomethyl ether 2000 diethanolamine (mPEG$_{2K}$DEA) (**S5**)*

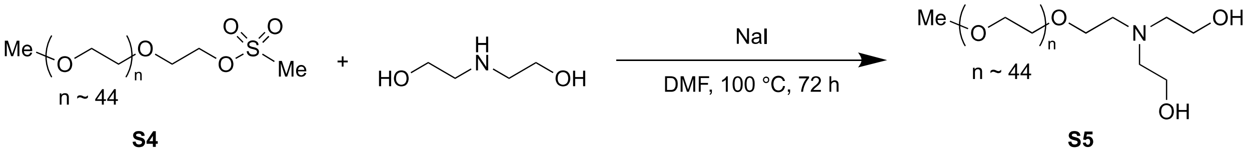

Compound **S4** (3.57 g, 1.79 mmol, 1 eq.) was dried under reduced pressure with heating at 60 °C for 1 h in a 100 mL round bottom flask. Once dried, **S4** was cooled to room temperature and dissolved in anhydrous DMF (15 mL) under nitrogen. Sodium iodide (0.274 g, 1.83 mmol, 1 eq.)

was added to the vessel and the reaction mixture was heated to 60 °C and allowed to stir for 10 minutes under nitrogen producing a dark red solution. Diethanolamine (1.31 g, 12.4 mmol, 6.8 eq.) was added dropwise to the reaction vessel under nitrogen. The resulting dark yellow reaction mixture was heated to 100 °C and allowed to stir under nitrogen for 72 h. The reaction mixture was cooled to room temperature and the solvent was removed under reduced pressure to yield the crude product as a dark red syrup. The crude product was dissolved in chloroform (50 mL) and washed with a saturated bicarbonate solution (1X 60 mL). The aqueous layer was then back extracted with chloroform (3X 60 mL) and the combined organic layers were dried over sodium sulfate and filtered. A majority of the solvent was then removed under reduced pressure to yield the crude product as a dark red syrup. The crude product was precipitated by the addition of cold ether (10X v/v). The precipitate slurry was incubated at – 20 °C for 16 h. The pure product was isolated using vacuum filtration and washed with additional cold ether. Product was immediately transferred to a vial and placed under vacuum to dry to completeness yielding the compound **S5** as a white powder (2.83 g, 73.2% yield, 100% functionalization).

^{1}H NMR (700 MHz, $CDCl_3$) δ: 3.65 (br, 208H), 3.38 (s, 3H), 2.74 (t, 2H, J = 7.0 Hz), 2.69 (t, 4H, J = 7.0 Hz)

*3.1.6. Synthesis of polyethylene glycol monomethyl ether 2000 chloroethylamine (mPEG$_{2K}$CEA) (**S6**)*

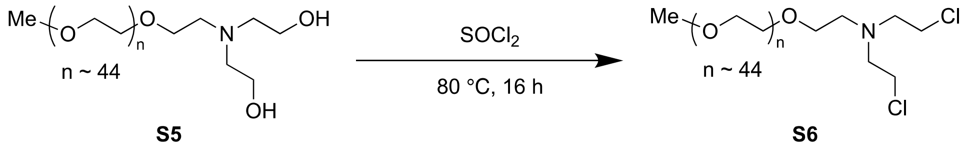

S5 **S6**

In a 50 mL round bottom flask equipped with a stir bar, compound **S5** (2.31 g, 2.8 mmol, 1 eq.) was dissolved in thionyl chloride (4 mL, 6.56 g, 55.17 mmol, 47.2 eq.). The resulting yellow reaction mixture was the refluxed at 80 °C for 16 h. Upon cooling to room temperature, the reaction was diluted with toluene (20 mL) and the solvent was removed under reduced pressure. Excess thionyl chloride was removed through co-evaporation with toluene (3X 20 mL) to yield the crude product was a dark orange syrup. The crude product was dissolved in chloroform (50 mL) and washed with a saturated bicarbonate solution (1X 60 mL). The aqueous solution was then back extracted with chloroform (3X 60 mL) and the combined organic layers were dried over sodium

sulfate and filtered. A majority of the solvent was then removed under reduced pressure to yield the crude product as a dark red syrup. The crude product was precipitated by the addition of cold ether (10X v/v). The precipitate slurry was incubated at – 20 °C for 16. The pure product was isolated using vacuum filtration and washed with additional cold ether. Product was immediately transferred to a vial and placed under vacuum to dry to completeness yielding the compound **S3** a beige powder (2.21 g, 88.5% yield, 88.5% functionalization).

^{1}H NMR (700 MHz, $CDCl_3$) δ_{ppm}: 3.65 (br, 215H), 3.38 (s, 3H), 2.95 (t, 4H, J = 7.0 Hz), 2.82 (t, 2H, J = 7.0 Hz)

*3.1.7. Synthesis of polyethylene glycol monomethyl ether 5000 mesylate (mPEG$_{5K}$OMs) (**S7**)*

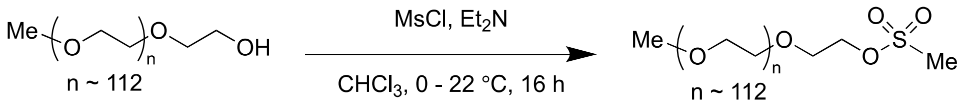

mPEG$_{5K}$ **S7**

To a 100 mL round bottom flask equipped with a stir bar, monomethyl ether polyethylene glycol (mPEG$_{5K}$, M_W = 5000 g/mol, 5.0002 g, 1 mmol, 1 eq.) was added and dissolved in chloroform ($CHCl_3$, 10 mL). Triethylamine (Et_2N, 0.512 g, 5.1 mmol, 5.1 eq.) was then added to the solution. The reaction was cooled to 0 °C using an ice bath and allowed to stir for 10 min. Methanesufonyl chloride (MsCl, 0.54 g, 4.7 mmol, 4.7 eq.) was added dropwise to the vessel. The reaction was allowed to warm to room temperature and stirred for 16 h. The reaction mixture was transferred to a separatory funnel and diluted with chloroform (90 mL) before being washed with 1 M HCl (1X 100 mL). The acidic aqueous layer was back extracted with chloroform (1X 100 mL) and the combined organic layers were washed with brine (1X 200 mL). The aqueous layer was back extracted with chloroform (1X 100 mL) and the combined organic layers were dried over sodium sulfate and filtered. A majority of the solvent was then removed under reduced pressure to yield the crude product as a dark orange syrup. The crude product was precipitated by the addition of cold ether (10X v/v). The precipitate slurry was incubated at – 20 °C for 16. The pure product was isolated using vacuum filtration and washed with additional cold ether. Product was immediately transferred to a 100 mL round bottom flask and placed under vacuum to dry to completeness yielding the compound **S7** as a white powder (3.28 g, 64% yield, 100% functionalization).

^{1}H NMR (700 MHz, $CDCl_3$) δ: 4.39 – 4.37 (m, 4H), 3.65 (br, 560H), 3.38 (s, 3H), 3.09 (s, 3H)

*3.1.8. Synthesis of polyethylene glycol monomethyl ether 5000 diethanolamine (mPEG$_{5K}$DEA) (**S8**)*

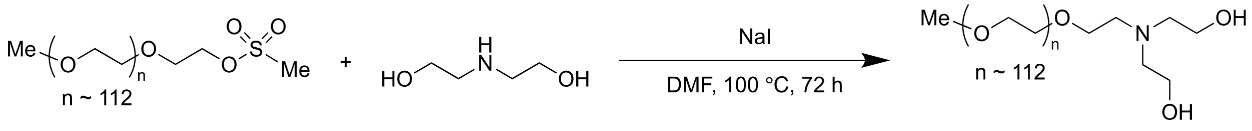

Compound **S7** (0.9776 g, 0.19 mmol, 1 eq.) was dried under reduced pressure with heating at 60 °C for 1 h in a 25 mL round bottom flask. Once dried, **S4** was cooled to room temperature and dissolved in anhydrous DMF (2 mL) under nitrogen. Sodium iodide (0.0706 g, 0.47 mmol, 1 eq.) was added to the vessel and the reaction mixture was heated to 60 °C and allowed to stir for 10 minutes under nitrogen producing a dark red solution. Diethanolamine (0.218 g, 2.1 mmol, 10.8 eq.) was added dropwise to the reaction vessel under nitrogen. The resulting dark yellow reaction mixture was heated to 100 °C and allowed to stir under nitrogen for 72 h. The reaction mixture was cooled to room temperature and the solvent was removed under reduced pressure to yield the crude product as a dark red syrup. The crude product was dissolved in chloroform (50 mL) and washed with a saturated bicarbonate solution (1x 60 mL). The aqueous layer was then back extracted with chloroform (3X 60 mL) and the combined organic layers were dried over sodium sulfate and filtered. A majority of the solvent was then removed under reduced pressure to yield the crude product as a dark red syrup. The crude product was precipitated by the addition of cold ether (10X v/v). The precipitate slurry was incubated at – 20 °C for 16 h. The pure product was isolated using vacuum filtration and washed with additional cold ether. Product was immediately transferred to a vial and placed under vacuum to dry to completeness yielding the compound **S8** as a white powder (0.66 g, 67% yield, 100% functionalization).

^{1}H NMR (700 MHz, $CDCl_3$) δ: 3.65 (br, 544H), 3.38 (s, 3H), 2.74 (t, 2H, J = 7.0 Hz), 2.69 (t, 4H, J = 7.0 Hz)

*3.1.9. Synthesis of polyethylene glycol monomethyl ether 5000 chloroethylamine (mPEG$_{5K}$CEA) (**S9**)*

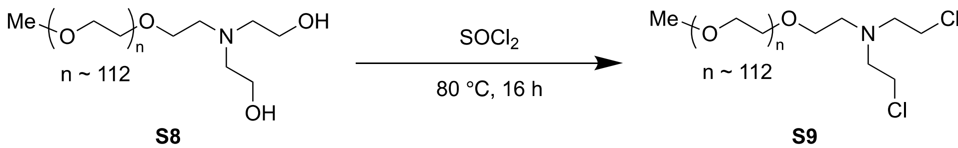

In a 25 mL round bottom flask equipped with a stir bar, compound **S8** (0.66 g, 0.13 mmol, 1 eq.) was dissolved in thionyl chloride (1 mL, 1.64 g, 13.8 mmol, 108 eq.). The resulting yellow reaction mixture was the refluxed at 80 °C for 16 h. Upon cooling to room temperature, the reaction was diluted with toluene (10 mL) and the solvent was removed under reduced pressure. Excess thionyl chloride was removed through co-evaporation with toluene (3X 10 mL) to yield the crude product was a dark orange syrup. The crude product was dissolved in chloroform (50 mL) and washed with a saturated bicarbonate solution (1X 60 mL). The aqueous solution was then back extracted with chloroform (3X 60 mL) and the combined organic layers were dried over sodium sulfate and filtered. A majority of the solvent was then removed under reduced pressure to yield the crude product as a dark red syrup. The crude product was precipitated by the addition of cold ether (10X v/v). The precipitate slurry was incubated at – 20 °C for 16. The pure product was isolated using vacuum filtration and washed with additional cold ether. Product was immediately transferred to a vial and placed under vacuum to dry to completeness yielding the compound **S9** a beige powder (0.44 g, 67% yield, 87.5% functionalization).

^{1}H NMR (700 MHz, $CDCl_3$) δ: 3.65 (br, 192H), 3.38 (s, 3H), 2.95 (br, 4H), 2.82 (br, 2H)

3.2. Synthesis of DNA BBPs

DNA BBPs were synthesized by reacting linear or cyclic DNA (pUC19 or pEYFP) with mPEG-CEA at a concentration of 0.1 mg/mL (small-scale) or 1 mg/mL (large-scale) at 37 °C for 1 – 1.5 h. Alkylators were used in excess based on µmole of nucleotides in each reaction. Success of alkylation was then characterized using agarose gel electrophoresis. Large-scale samples were purified using Amicon Ultra spin columns (0.5 or 15 mL) with a 10 kDa MWCO to remove unreacted excess mPEG – CEA. An example calculation of mass of alkylator needed for a 2 µg sample of pEYFP is shown below.

Example: 2 µg of pEYFP (5.045 kb) with 20 equivalents of $mPEG_{2k}CEA$

$$\mu moles\ of\ nucleotides = \frac{2\ \mu g\ pEYFP}{3112765 \frac{\mu g\ pEYFP}{\mu mol\ pEYFP}} * \frac{5045\ \mu mol\ bp}{1\ \mu mol\ pEYFP} * \frac{2\ \mu mol\ nucleotides}{1\ \mu mol\ bp} = 6.48\ x\ 10^{-3}\ \mu mol$$

$$Mass\ of\ mPEG_{2K}CEA = 6.48\ x\ 10^{-3} * 20 * \frac{1\ \mu mol\ mPEG_{2K} - CEA}{2\ \mu mol\ CEA} * 2138 \frac{\mu g\ mPEG_{2K} - CEA}{\mu mol\ mPEG_{2K} - CEA} = 139\ \mu g$$

Ring pEYFP (4.1 µL of a 0.4936 µg/µL solution, 2 µL, 6.48 x 10^{-3} µmol) was added to a sterile 1.5 mL Eppendorf tube and diluted with of 1X TAE buffer, pH 8.3 (14.7 µL). $mPEG_{2K}CEA$ (23.6 mg, 11 µmol) was dissolved in 221 µL of 1X TAE buffer to obtain a fresh 107 mg/mL solution. 1.3 µL (139 µg, 6.48 x 10^{-3} µmol, 20 eq.) of the solution was added to the Eppendorf tube containing pEYFP. The mixture was thoroughly mixed and then centrifuged before being placed on a heating block and incubated at 37°C for 1 hour. Success of alkylation was assessed using agarose gel electrophoresis to determine shift is apparent molecular weight compared to unmodified solutions.

3.3. Small-scale relaxation of pUC19

10X NEBuffer r3.1 (2 µL), Nt. BspQI (2 µL, 1 U/µg pDNA), and nuclease-free water (41.7 µL) were added to a sterile 1.5 mL Eppendorf tube. pUC19 (1.3 µL, 1 µg) was added and the reaction tube was gently vortexed for 3 s and then centrifuged at max speed for 15 s. Restriction digest was incubated at 50 °C for 1 h. After success of digest was confirmed using AGE, EDTA (2 µL, 0.5 M) was added to a final concentration of 5 mM to inactivate Nt. BspQI.

3.4. Small-scale linearization of pUC19

10X EcoRI buffer (2 µL), EcoRI (2 µL, 1 U/µg pDNA), and nuclease-free water (16.7 µL) were added to a sterile 1.5 mL Eppendorf tube. pUC19 (1.3 µL, 1 µg) was added and the reaction tube was gently vortexed for 3 s and then centrifuged at max speed for 15 s. Restriction digest was incubated at 37 °C for 1 h. After success of digest was confirmed using AGE, EDTA (0.8 µL, 0.5 M) was added to a final concentration of 5 mM to inactivate EcoRI.

3.5. Large-scale linearization of pEYFP

The following protocol was adapted from Tellez and Cole.[1] Nuclease-free water (1086 µL), 10X EcoRI buffer (650 µL), and BamHI (414 µL, 0.2 U/µg pDNA) were added to a 15 mL conical tube containing 4.4 mL of pEYFP (4.865 mg/mL, 21.4 mg) for a total volume of 6.5 mL. Solution was mixed by gently inverting and pipetting up and down. Restriction digest was incubated at rom temperature for 16 h. After success of digest was confirmed using AGE, EDTA (65 µL, 0.5 M) was added to a final concentration of 5 mM to inactivate EcoRI. 1 volume of isopropanol was added, and the tube was inverted gently 6x. DNA should precipitate immediately. Solution was incubated at room temperature for 2 h before DNA was harvested by centrifugation (12000 g, 40 min, 4 °C). Pellet was washed with 70% ethanol and reformed by centrifugation (12000 g, 15 min, 4 °C). The pellet was dried and transferred to a sterile Eppendorf tube using a sterile 20G needle. 1 mL of sterile 1X TAE pH 8.3 was added to the tube and DNA was incubated at 4 °C to dissolve over the weekend. Success of digests was determined using AGE.

3.6. Agarose gel electrophoresis (AGE)

Typically, 0.5 g of agarose (Top Vision) was suspended in 100 mL of 1X TAE buffer (0.5%). The solution was then heated in a microwave until it boiled (approximately 5 minutes) before it was allowed to cool to room temperature. After cooling, 3.5 µL of an ethidium bromide solution (10 mg/mL) was added. The solution was then poured into a 12 cm 20-well gel and left to solidify for approximately 30 minutes. The gel was submerged in 1X TAE buffer and the comb was removed. For the ladder, 0.25 µg of GeneRuler 1 kb ladder was loaded while 0.05 µg of DNA was loaded for standards and samples for DNA BBPs. For DNase I activity tests, 0.1 µg of standards and samples were loaded to better visualize DNA degradation.

3.7. UV-Vis measurements before and after alkylation

Table S1. Absorbance of DNA solution at 260 nm before and after reacting with $mPEG_{750}CEA$, $mPEG_{2K}CEA$, or $mPEG_{5K}CEA$.

mPEG-CEA	Volume of mPEGCEA (µL)	Volume of linear DNA (µL)	Volume of 1X TAE Buffer (µL)	A_{260} Before	A_{260} After
750	2.16	6	16.8	0.49	0.59
2000	5.20	6	13.8	0.53	0.47
5000	3.12	6	15.9	0.60	0.66

3.8. DNA mica deposition protocol

Samples were diluted to 1 ng/µl with buffer (40 mM HEPES (pH 6.6-6.8), 5mM $NiCl_2$,) using HCl to adjust the pH. A 10 mm diameter mica (Ted Pella, 50) affixed to a magnetic puck (PELCO AFM Workstation) was freshly cleaved with Scotch tape. We then placed 50 µL of sample dropwise onto the freshly cleaved mica.

Samples were diluted to 1 ng/µL with sterile filtered DI water. A 10 mm diameter mica disc (Ted Pella, 50) attached to a magnetic puck (PELCO AFM Workstation) was freshly cleaved with Scotch tape. Then, 50 µL of Poly-L-ornithine was applied dropwise onto the freshly cleaved mica and allowed to incubate for 1 minute. The mica was rinsed with 1 mL of sterile filtered DI water and dried under a stream of nitrogen gas. After diluting samples to 1 ng/µL with sterile filtered DI water, 50 µL of the sample was added dropwise and incubated for 1 minute. The mica was rinsed again with 1 mL of sterile filtered DI water, ensuring it remained moist before imaging.

3.9. AFM imaging

All samples were imaged using a commercial AFM (Cypher ES, Asylum Research), equipped with a temperature-controlled, closed fluidic holder. The sample temperature was maintained at 20°C. An AC240TSA-R3 cantilever with a frequency of 70 kHz and a spring constant of 2 N/m was utilized. Fifty microliters of buffer were applied to the cantilever tip, which was then carefully inverted upon placement into the cantilever holder to prevent snapping due to capillary action. The cantilever was tuned and driven using blueDrive Photothermal excitation.

Images were obtained in tapping mode, targeting an amplitude of 70 mV. To approach the surface, the setpoint was adjusted to approximately 80% of the target amplitude (about 63 mV), and the tip was lowered at a rate of 2 μm/s. It was re-tuned as necessary until making contact with the mica surface. Prior to imaging, the samples and cantilever were incubated for 30 minutes. The integral gain was set at 110, with the initial setpoint approximately 10% higher than the target amplitude (around 77 mV). The setpoint was carefully lowered to achieve desirable tracking. A constant scanning rate of 2 Hz was maintained for all images.

3.10. DNase I Activity Test

To six sterile 1.5 mL Eppendorf tubes, 1 μg of DNA or DNA-BBP was added followed by nuclease-free water (to total volume of 20 μL), and 2 μL of 10X DNase I reaction buffer with $MgCl_2$. DNase I was freshly diluted to 0.001 U/μL by combining 1 μL of DNase I with 999 μL of 1X DNase I reaction buffer $MgCl_2$. Diluted DNase I (2 μL, 0.0001 U/μL, 0.002 U/μg) was added to reaction tubes and incubated at 22 °C for either 5, 10, 20, 40, or 60 min. Enzyme was added to longest incubation time first to ensure samples were not sitting for extended periods of time before analysis. Reaction digests were terminated by the addition of 1 μL of 0.5 M EDTA to inactivate the enzyme. For negative controls, DNA or DNA-BBPs were incubated in 1X DNase I reaction buffer for 60 min in the absence of enzyme to confirm that any degradation is due to exposure to DNase I alone and not some other reaction condition. AGE was used to analyze the extent of degradation.

4. Supporting figures

4.1. Agarose gels

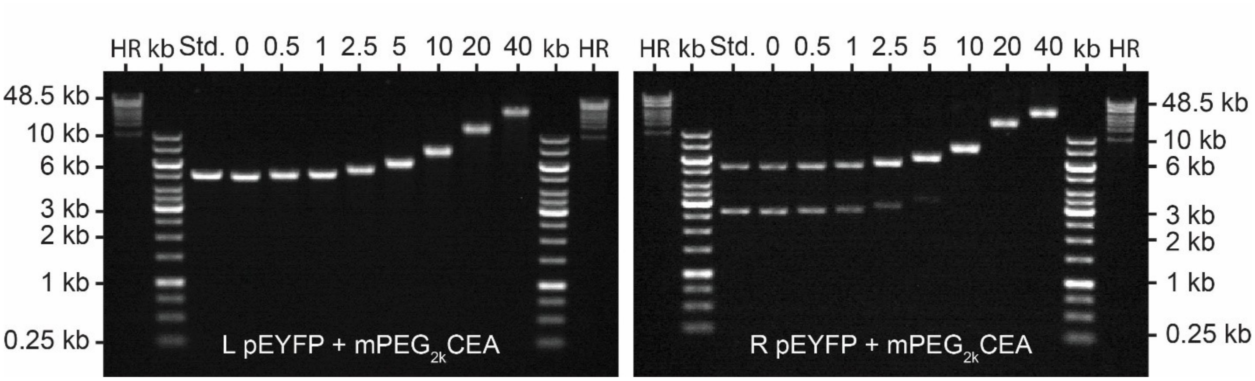

Figure S1. *Electrophoresis analysis for alkylation experiments of L (left) and R (right) pEYFP (5045 bp) with mPEG2KCEA on a 0.5% agarose gel. pEYFP was reacted with various concentrations of mPEG2KCEA for 1.5 h at 37 °C to yield R-BBPs and L-BBPs. HR stands for the GeneRuler High Range molecular weight ladder and kb stands for the GeneRuler 1 kb molecular weight ladder. The standard was either unfunctionalized linear or ring pEYFP. The numbers at the top of each gel represent the equivalents of mPEGCEA added relative to the number of nucleotides in each sample.*

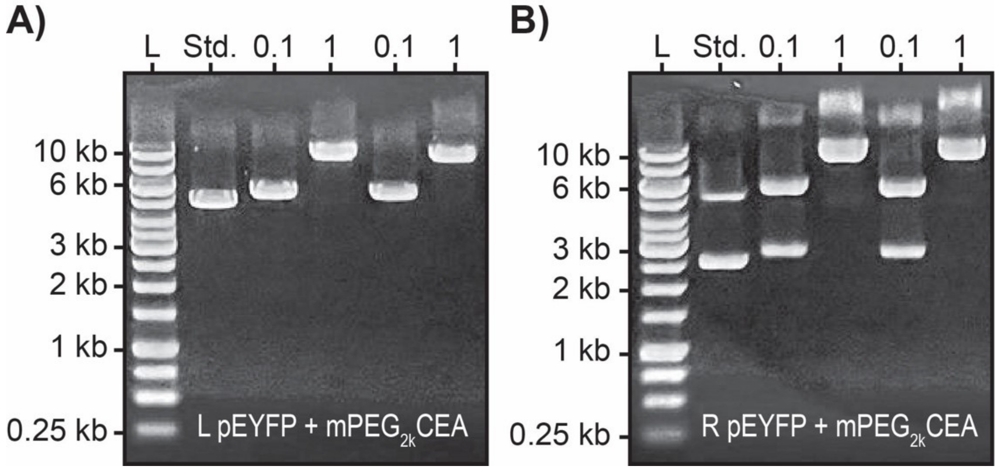

Figure S2. *Gel electrophoresis analysis of pEYFP reacting with 2 eq. of mPEG$_{2K}$CEA at different concentrations on a 0.5% agarose gel. A) 20 µg of linear pEYFP reacted with 2 eq. of mPEG$_{2K}$CEA at 0.1 and 1 mg/mL. B) 20 µg of ring pEYFP reacted with 2 eq. of mPEG$_{2K}$CEA at 0.1 and 1 mg/mL. L stands for the GeneRule 1 kb molecular weight ladder and the standard was unfunctionalized L orR pEYFP. The numbers at the top of each gel represent the concentration of DNA in each reaction.*

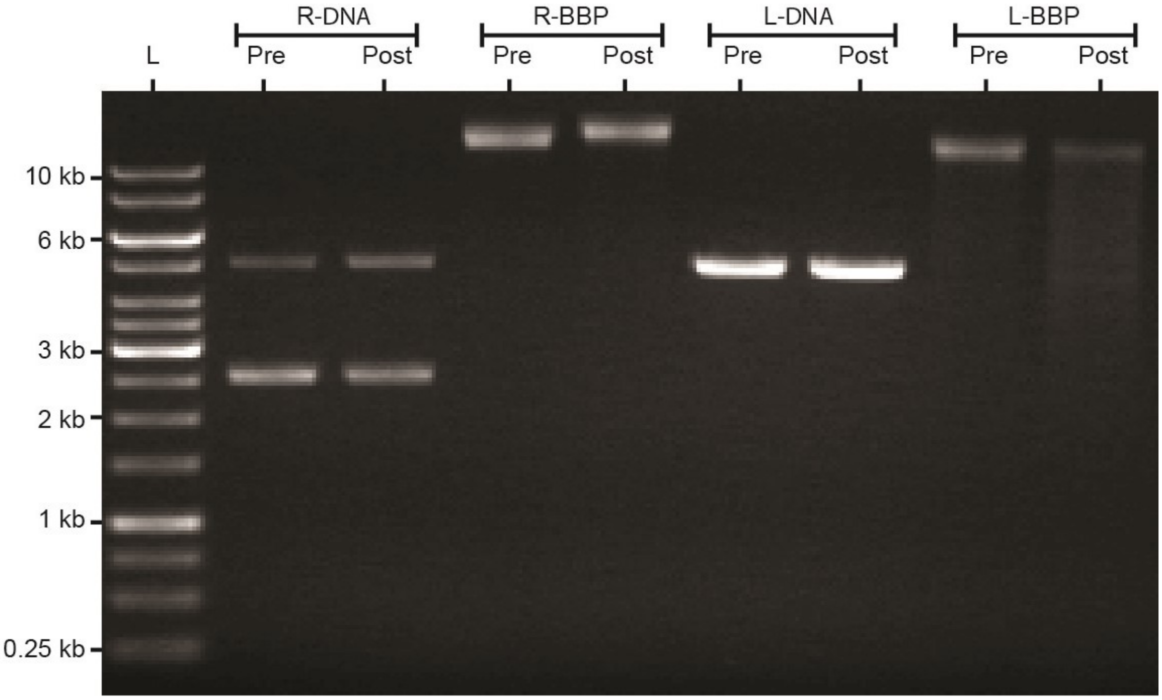

Figure S3. *Gel electrophoresis analysis of pre and post rheology samples on a 0.5% agarose gel. Lane 1: GeneRuler 1 kb molecular weight ladder, Lane 2: R-DNA (ring pEYFP) sample before rheology, Lane 3: R-DNA sample after rheology, Lane 4: 42.2% R-BBP (ring pEYFP BBP) sample before rheology, Lane 5: 42.2% R-BBP sample after rheology, Lane 6: L-DNA (linear pEYFP) sample before rheology, Lane 7: L-DNA sample after rheology, Lane 8: 40.1% L-BBP (linear pEYFP BBP) sample before rheology, Lane 9: 40.1% L-BBP sample after rheology.*

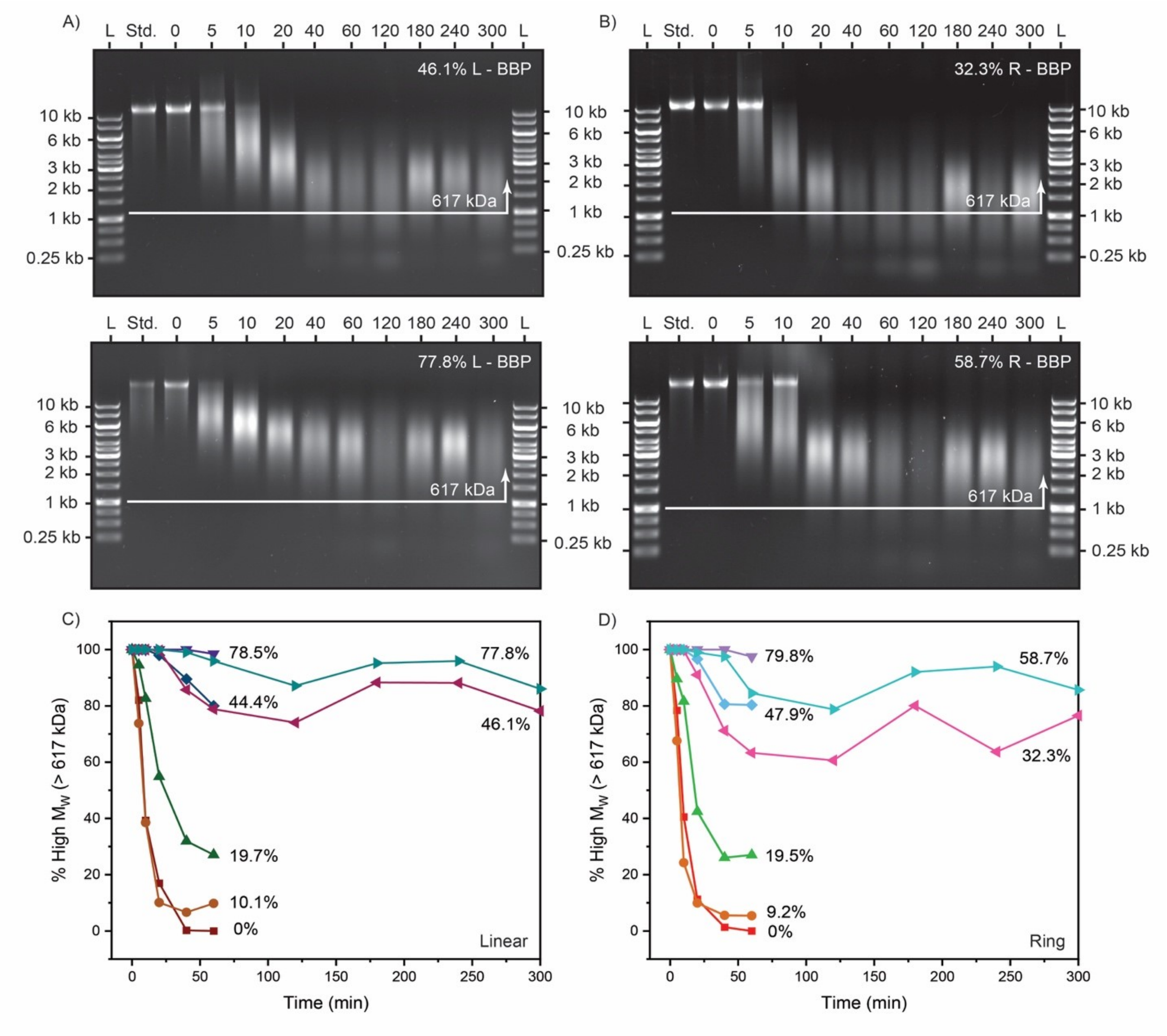

Figure S4. *Enzymatic stability tests of DNA BBPs using pEYFP and DNase I with extended incubation times for high graft density BBPs (> 20%) compared to original samples. A) Gel electrophoresis analysis of degradation of L-BBP conjugates on a 0.5% agarose gel. L-BBPs had graft densities of 46.1 and 77.8%. B) Gel electrophoresis analysis of R-BBP conjugates on a 0.5% agarose gel. R-BBPs had graft densities of 32.3 and 58.7%. For A) and B), L stands for the GeneRuler 1 kb molecular weight ladder and standards were samples of corresponding DNA BBP before exposure to reaction buffer or enzyme. The numbers at the top represent the incubation time in minutes for each digest at 22 °C. C) Percentage of higher molecular weight DNA (>1 kb or 617 kDa) of each L-BBP compared to unmodified L-DNA in the presence of DNase I as a function of time. D) Percentage of higher molecular weight DNA (> 1 kb or 617 kDa) of each R-BBP compared to unmodified R-DNA in the presence of DNase I as a function of time. For C) and D), the percentage of higher molecular weight DNA was determined by gel band densitometry analysis.*

4.2. Bulk oscillatory rheology

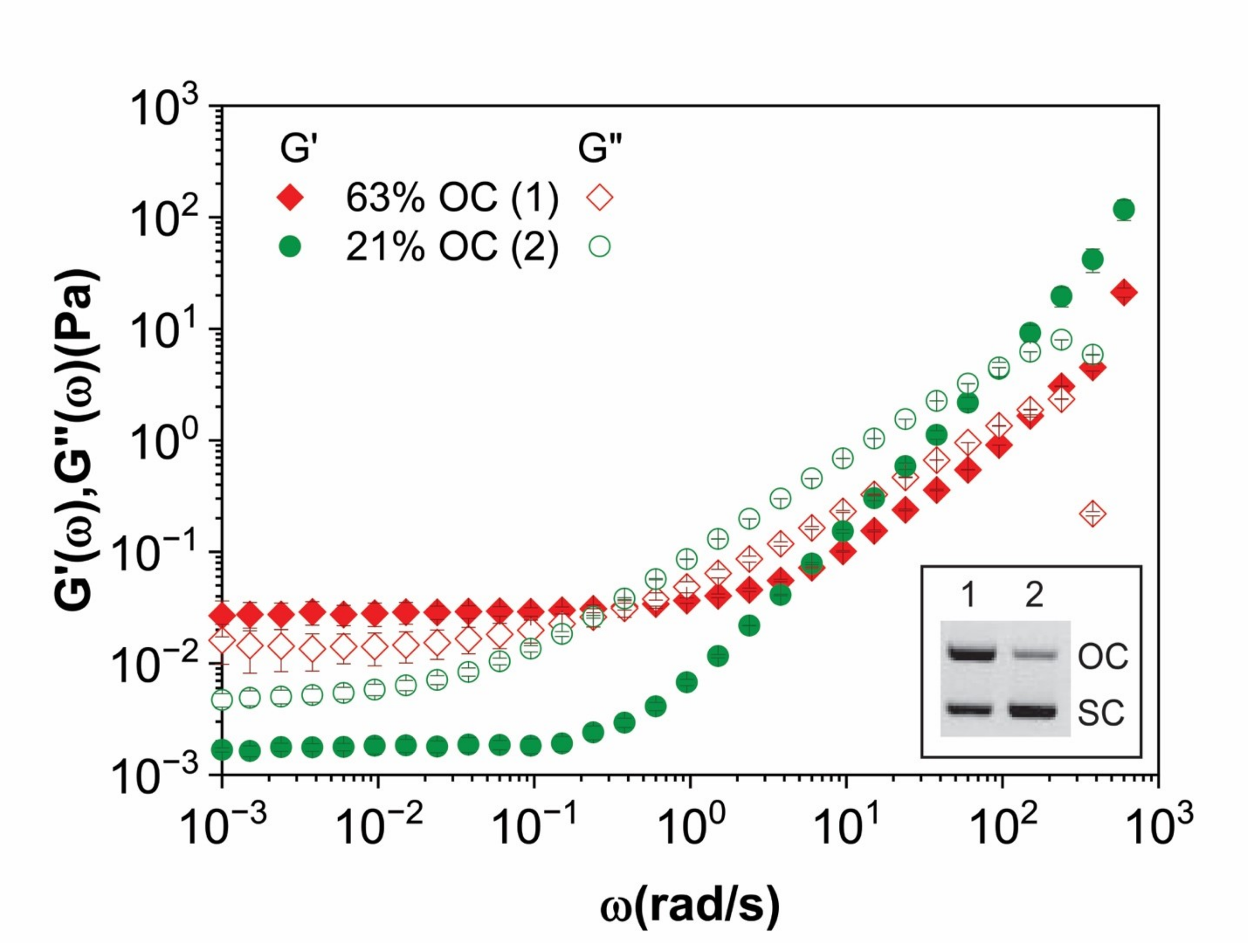

Figure S5. *Bulk linear oscillatory rheology measurements on 10 mg/mL solutions of pEYFP from different preparations. Linear viscoelastic moduli, G'(ω) (storage modulus, closed symbols) and G''(ω) (loss modulus, open symbols) versus angular frequency, ω, for pEYFP at 10 mg/mL using a 180 µL sample volume. Preparation 1 (prep 1 [1], red diamonds) contains 63% OC and 37% SC DNA while preparation 2 (prep 2 [2], green circles) contains 21% OC and 79% SC DNA. Data shown is an average of 2 independent measurements for prep 1 and 3 independent measurements for prep 2. The error bars represent standard errors. All measurements were performed on a TA Instruments DHR-3 rheometer with parallel plate geometry. Image depicted in bottom right shows agarose gel electrophoresis analysis of the samples used for the reported data highlighting the different isoforms (SC and OC) of pEYFP.*

4.3. NMR Spectra

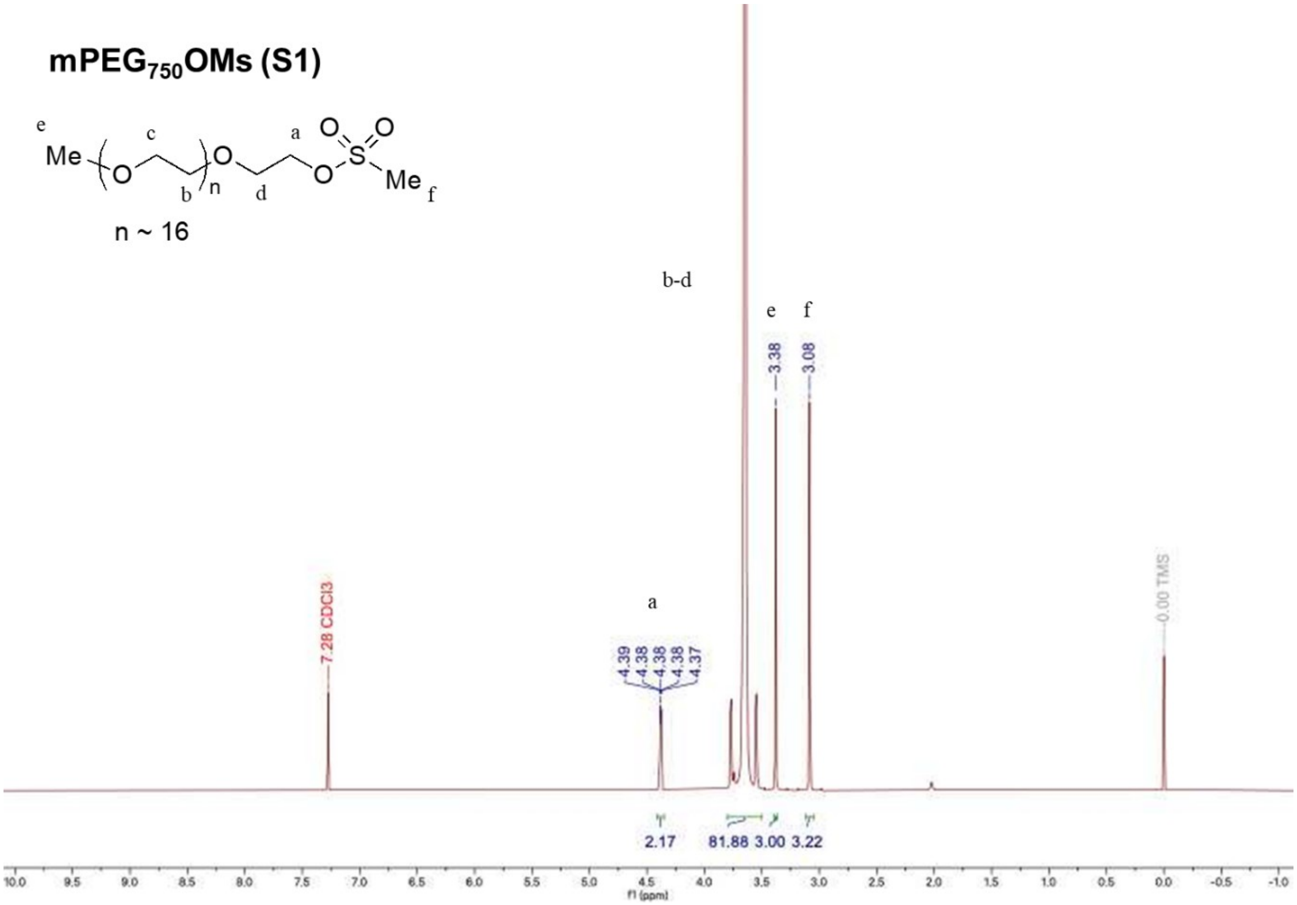

Figure S6. *^{1}H NMR ($CDCl_3$, 700 MHz) of mPEG$_{750}$OMs (**S1**).*

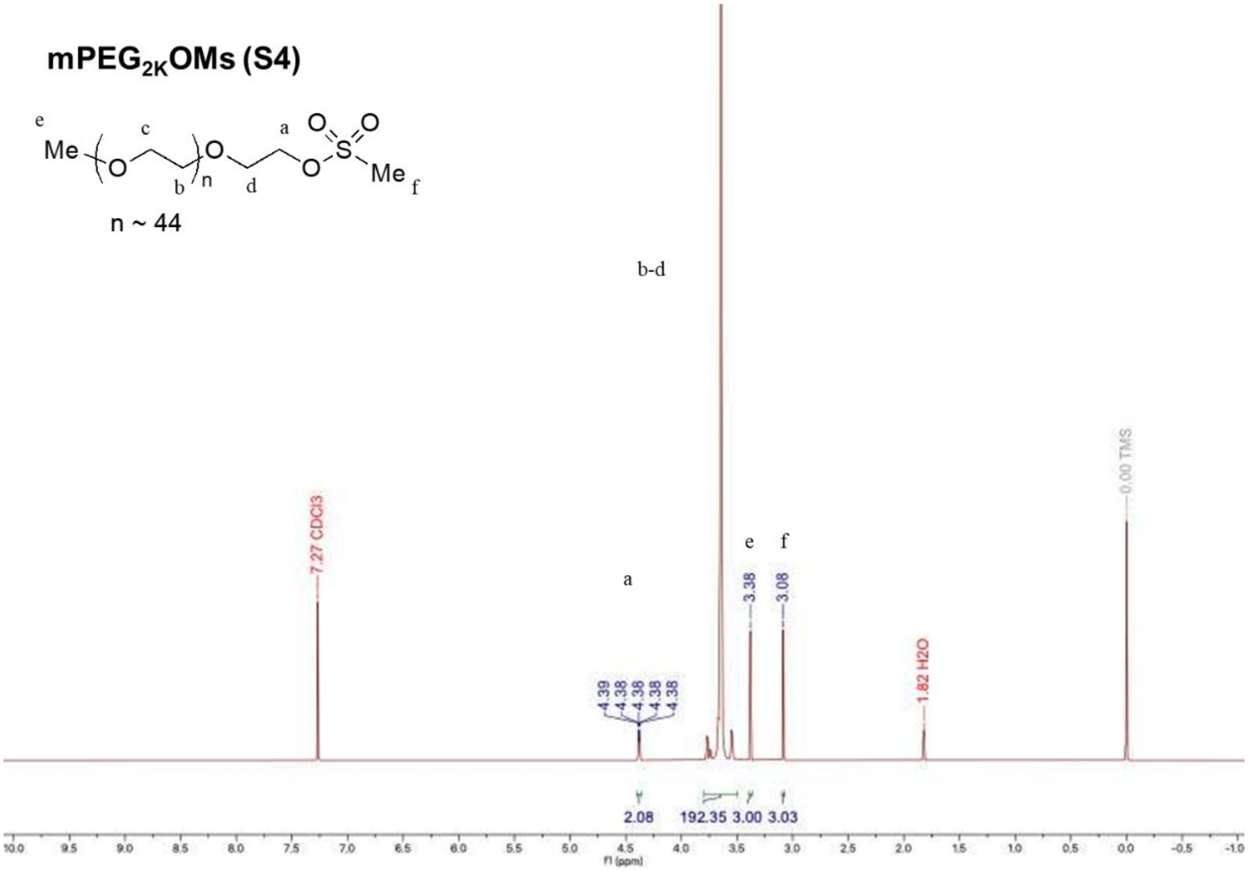

Figure S7. *^{1}H NMR ($CDCl_3$, 700 MHz) of mPEG$_{2K}$OMs (**S4**).*

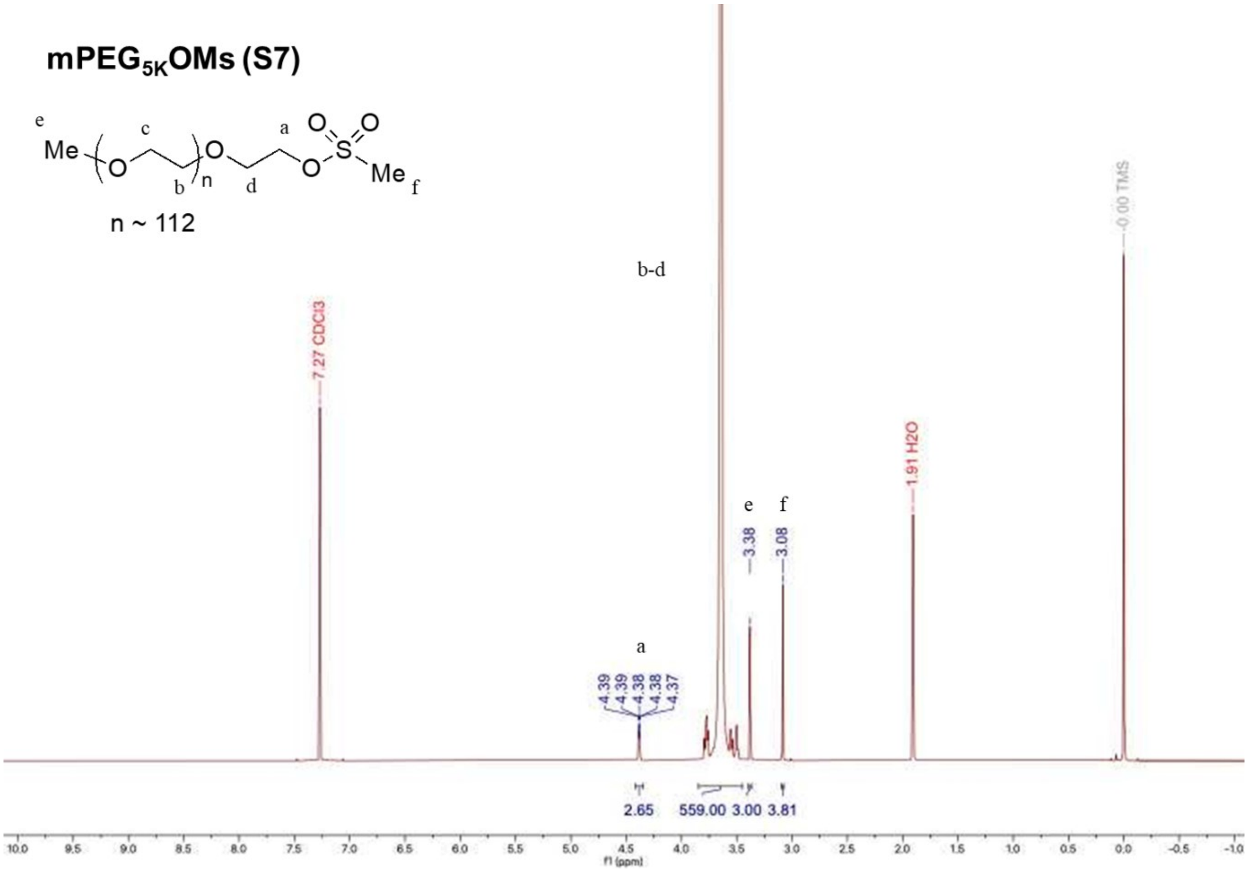

Figure S8. *^{1}H NMR (CDCl$_3$, 700 MHz) of mPEG$_{5K}$OMs (**S7**).*

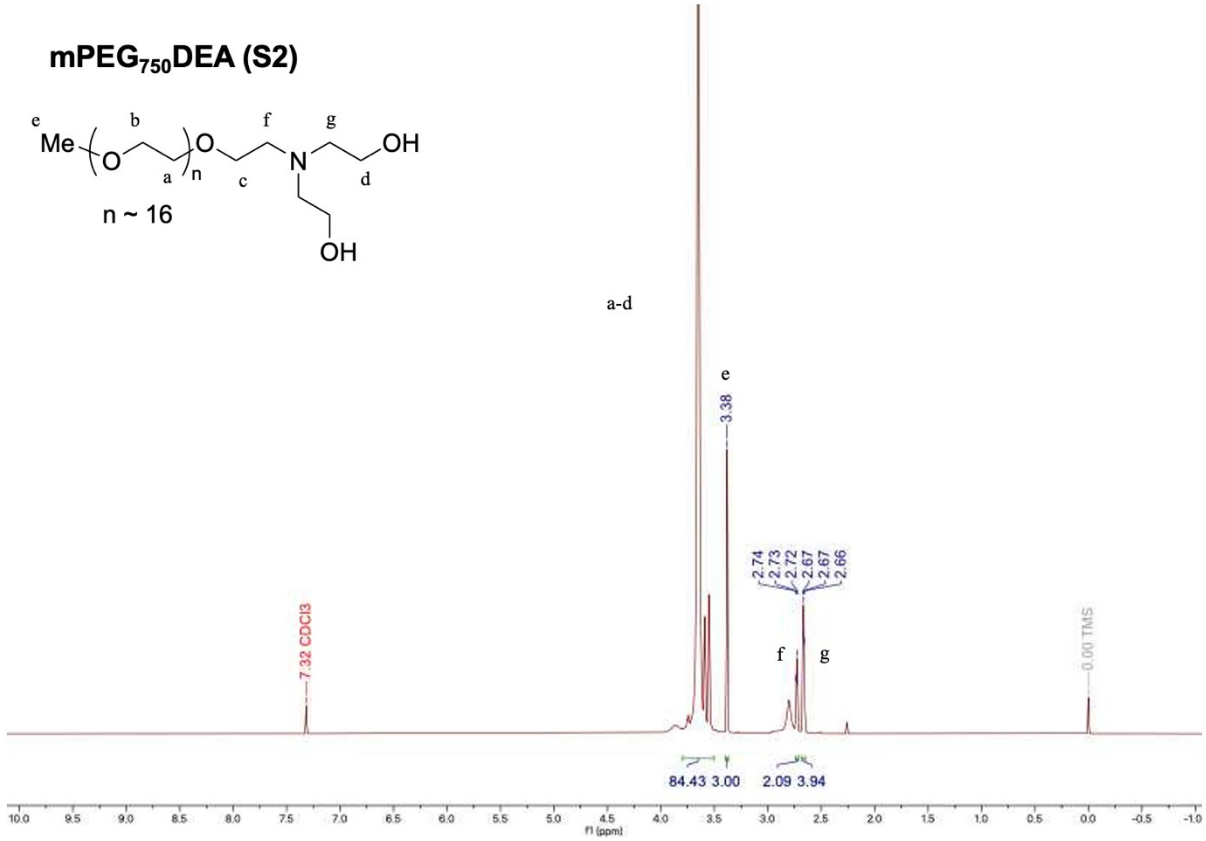

Figure S9. *^{1}H NMR (CDCl$_3$, 700 MHz) of mPEG$_{750}$DEA (**S2**).*

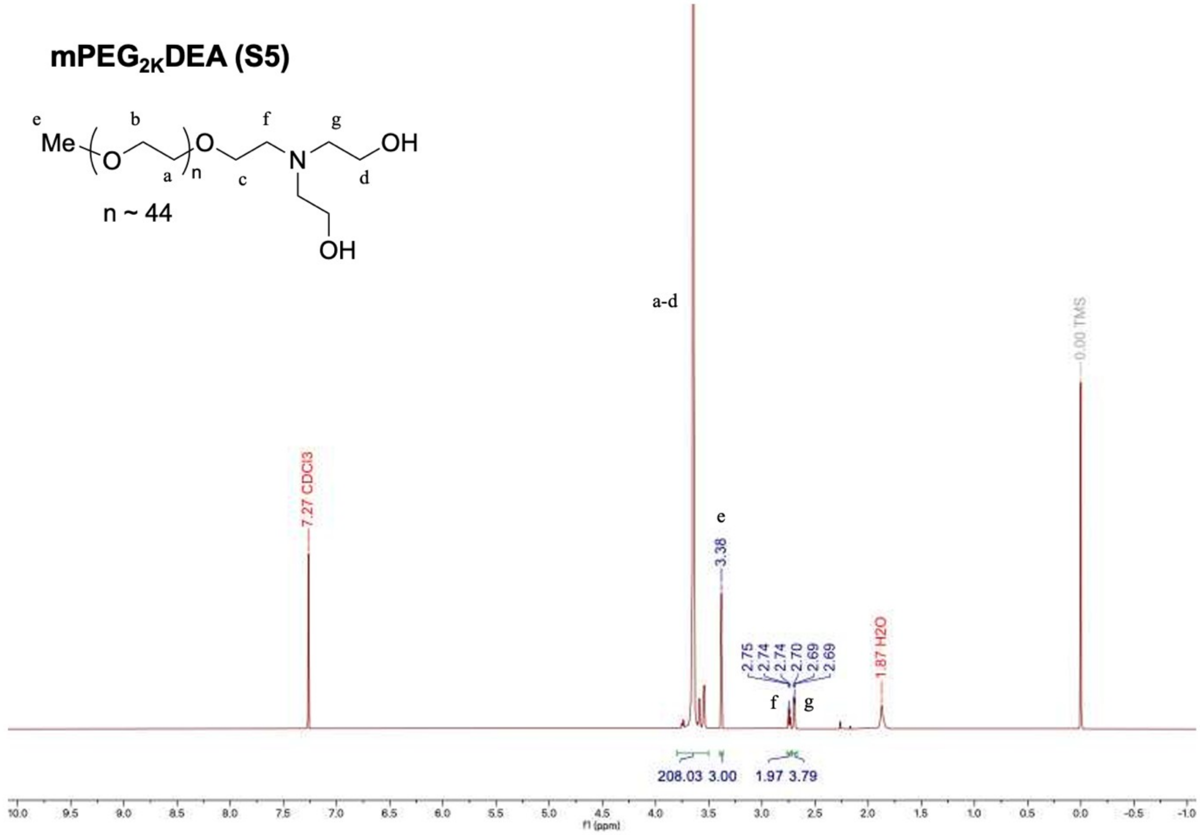

Figure S10. *^{1}H NMR (CDCl$_3$, 700 MHz) of mPEG$_{2K}$DEA (**S5**).*

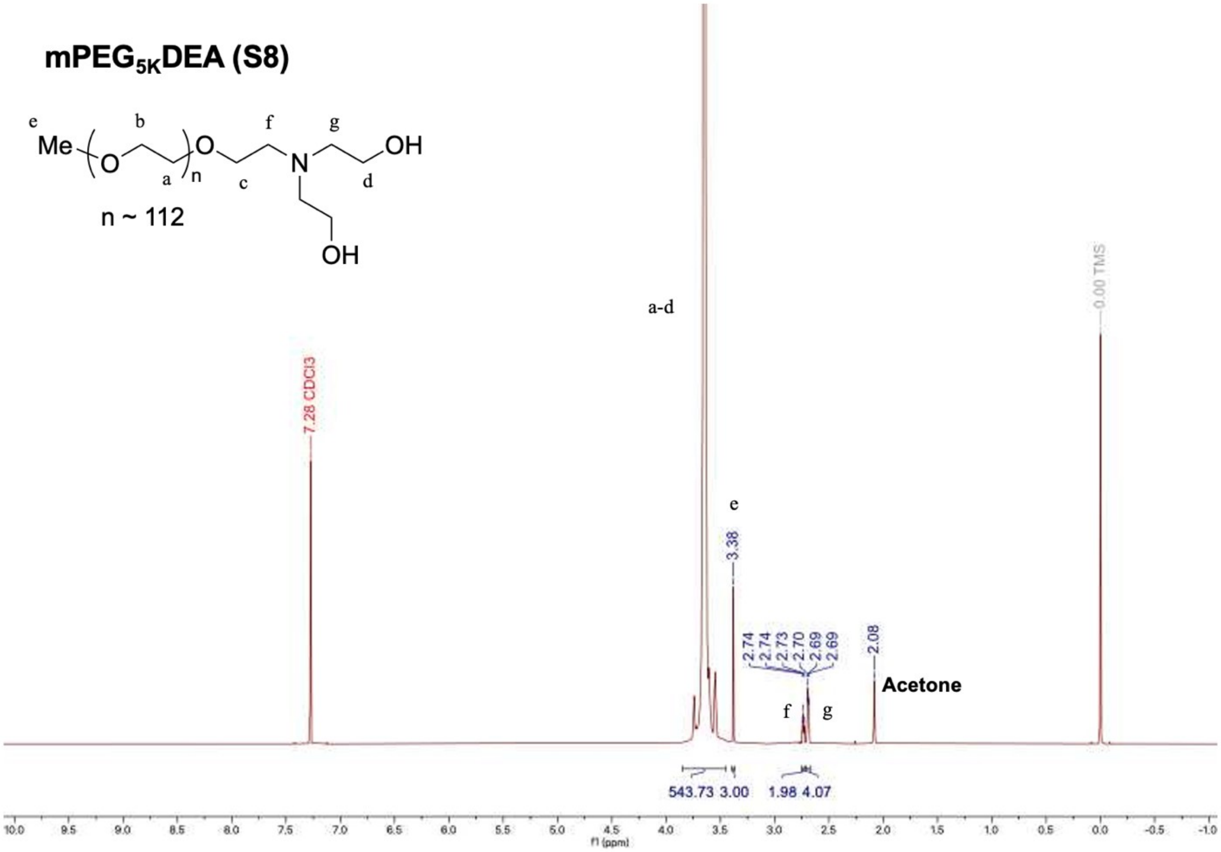

Figure S11. *^{1}H NMR ($CDCl_3$, 700 MHz) of mPEG$_{5K}$DEA (**S8**).*

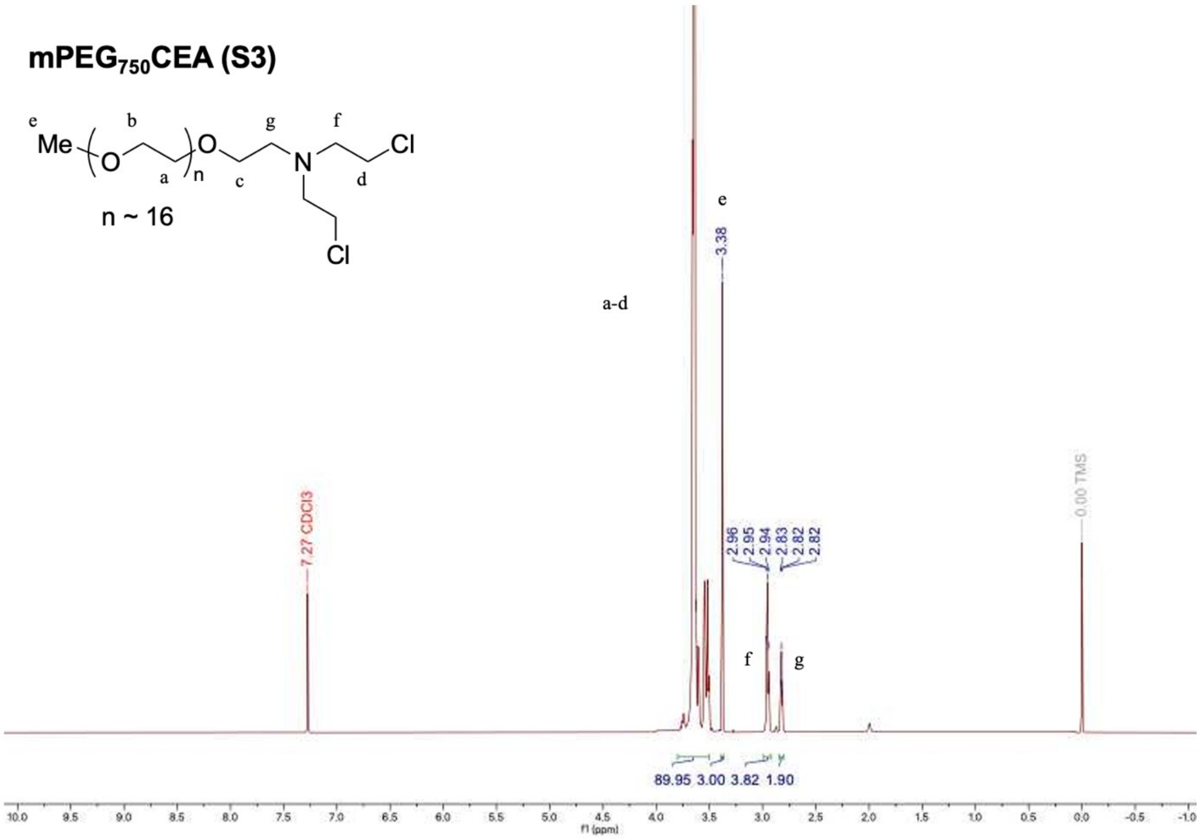

Figure S12. *^{1}H NMR (CDCl$_3$, 700 MHz) of mPEG$_{750}$CEA (**S3**).*

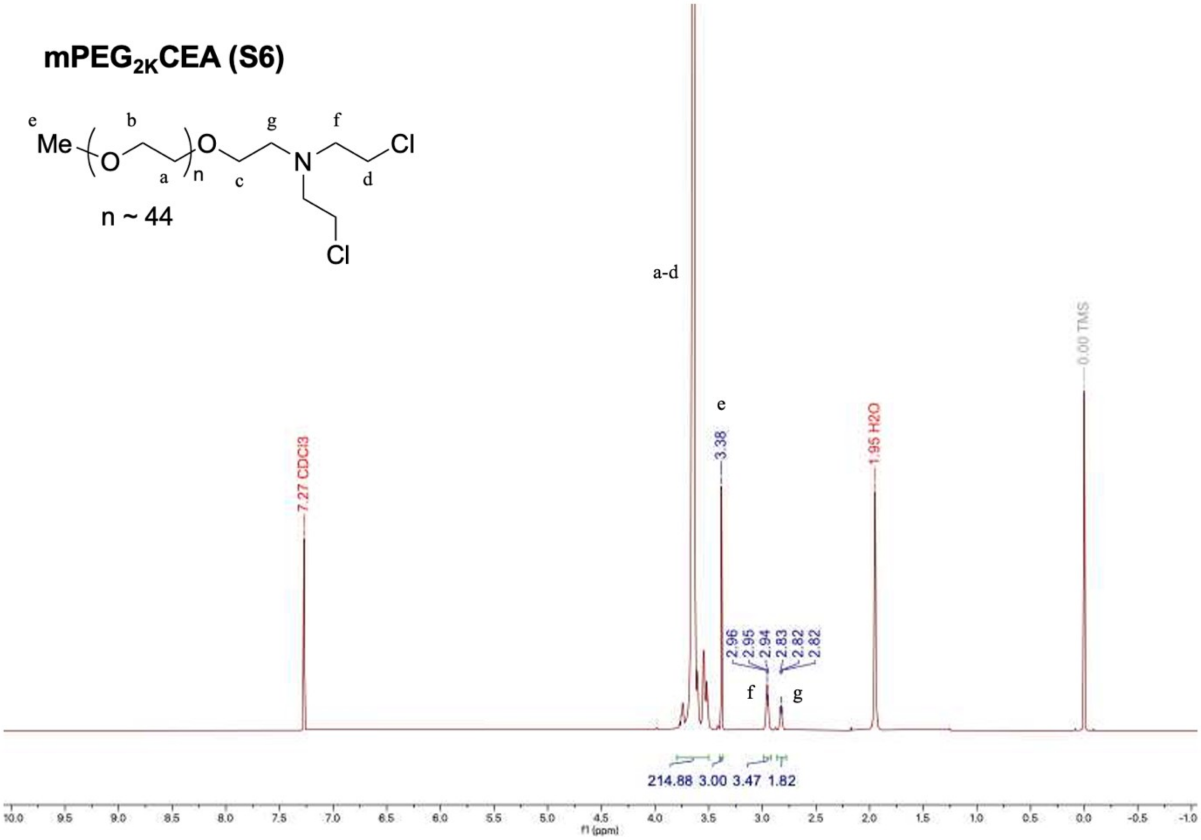

Figure S13. *^{1}H NMR ($CDCl_3$, 700 MHz) of mPEG$_{2K}$CEA (**S6**).*

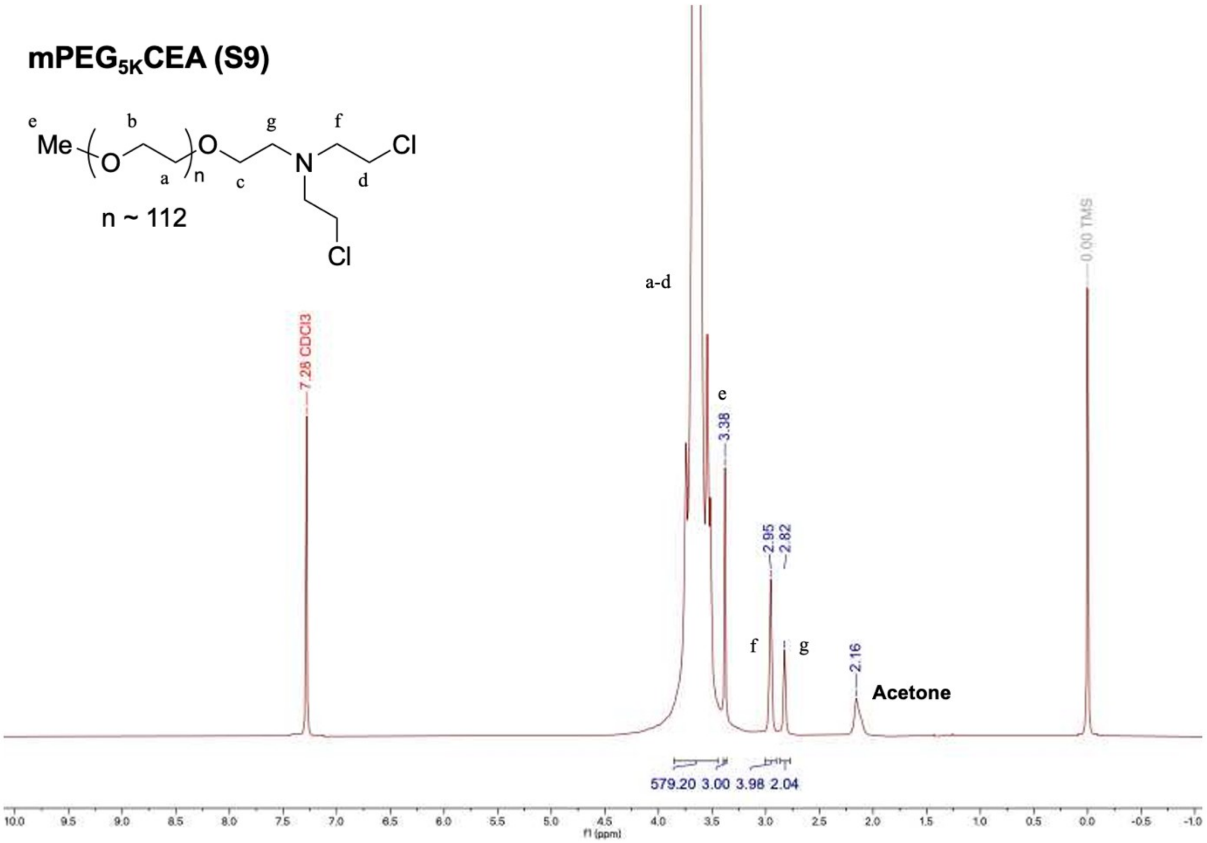

Figure S14. *^{1}H NMR ($CDCl_3$, 700 MHz) of mPEG$_{5K}$CEA (**S9**).*
